# Optical write–erase chemical memory state in plasmonic nanoarrays

**DOI:** 10.1039/d5sc07368e

**Published:** 2025-12-03

**Authors:** Victor Tabouillot, Muhammad Murad, Dylan Wilkinson, Rahul Kumar, Paula L. Lalaguna, Maryam Hajji, Affar S. Karimullah, Nikolaj Gadegaard, Aurélie Malfait, Patrice Woisel, Graeme Cooke, Malcolm Kadodwala

**Affiliations:** a School of Chemistry, Joseph Black Building, University of Glasgow Glasgow G12 8QQ UK malcolm.kadodwala@glasgow.ac.uk 2604448t@student.gla.ac.uk; b School of Engineering, Rankine Building, University of Glasgow Glasgow G12 8LT UK; c Univ. Lille, CNRS, INRAE, Centrale Lille, UMR 8207 – UMET – Unité Matériaux et Transformations F-59000 Lille France

## Abstract

Plasmonic nanoarrays coated with a thermoresponsive self-assembled monolayer (SAM) operate as an optically programmable write–erase chemical memory. Optical addressing through pulsed laser illumination writes a collapsed interfacial state, the SAM stores this state for days, and passive rehydration erases it to restore chemical functionality. Unlike previous PNIPAM systems, where laser-induced collapse is transient on nanosecond–minute timescales, the SAM forms a kinetically trapped, long-lived state that enables durable storage of chemical information. By tuning the wavelength and polarisation of uniform illumination, individual nanoarrays can be addressed selectively. Switching is detected through spectral shifts in the localised surface plasmon resonance and suppression of biomolecular binding, and supported by electromagnetic and transient thermal simulations based on measured nanostructure geometries. These results establish a general framework for multiplexed, optically programmable write–erase surface chemistry, opening routes to erasable nanofabrication strategies and multiplexed biosensing.

## Abstract 2

Plasmonic nanoarrays act as optical chemical memory: laser-induced PNIPAM collapse writes a persistent state, erased by passive hydration. Resonant, geometry-selective heating enables multiplexed, rewritable nanoscale functionality.

## Graphical abstract

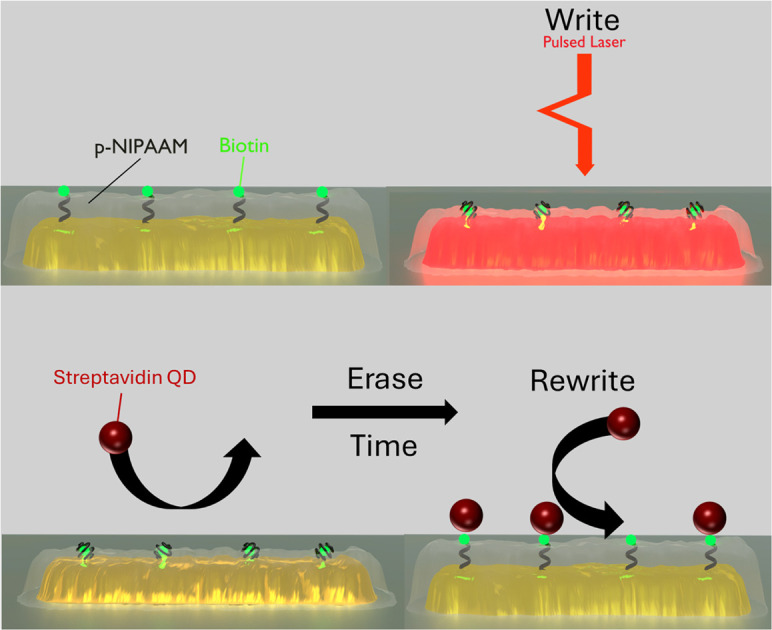

Plasmonic nanostructures convert light into strongly confined electromagnetic fields and localised heat, enabling control of interfacial chemistry on the nanoscale. Thermoplasmonics has established how absorption resonances and geometry determine nanoscale temperature fields and can be exploited for reactions, actuation, and switching.^1–3^ Despite this progress, existing strategies have not provided a way to write, store, and subsequently erase chemical functionality in a controllable fashion.

Thermoresponsive polymers—particularly poly(*N*-isopropylacrylamide) (PNIPAM)—undergo a reversible coil-to-globule transition above the lower critical solution temperature (LCST), producing pronounced changes in hydration, thickness, and surface accessibility.^4,5^ For surface-grafted formats, the collapse depends on molecular weight and grafting density and has been observed directly by AFM and QCM-D.^6,7^ These established behaviours provide the benchmark for our SAM-based system.

Under homogeneous thermal cycling, PNIPAM brushes collapse and rehydrate on laboratory timescales (<minutes); in contrast, when PNIPAM is driven by pulsed laser heating in core–shell nanoparticles, collapse and recovery can occur on the nanosecond timescale.^8^ Thus, while PNIPAM is highly responsive, prior systems generally exhibit short-lived collapsed states that limit long-term, programmable chemical control. Recent studies of thermoresponsive polymer–plasmonic hybrids have demonstrated optical modulation of PNIPAM-coated nanostructures on micro- to nanosecond timescales, highlighting the potential for light-driven, reversible polymer collapse in plasmonic environments.^9,10^

Here, we overcome this limitation. A PNIPAM self-assembled monolayer (SAM) can be driven into a collapsed state that persists for more than a day under aqueous conditions, below the LCST, before passively erasing. This orders-of-magnitude increase in lifetime transforms a transient thermal response into true chemical memory, where functionality can be written, stored, and later erased.

Unlike inorganic phase-change materials (PCMs), which rely on high-temperature amorphous–crystalline transitions (typically >673 K) and substantial thermal budgets, limiting their endurance and compatibility with soft-matter environments,^11,12^ the PNIPAM self-assembled monolayer (SAM) operates through a mild, fully reversible hydration–dehydration transition below 313 K. This low-energy, aqueous process eliminates structural fatigue and substrate damage, enabling rewritable, biocompatible operation. The monolayer format provides molecular-scale spatial precision and straightforward chemical tunability through end-group or copolymer modification. Direct coupling of the SAM to plasmonic resonances permits wavelength- and polarisation-selective optical addressing of chemical functionality—a capability not available in conventional PCM thin films.

Polymers are increasingly recognised as viable materials for information storage. Resistive and redox-active polymer devices exhibit non-volatile switching with low operating voltages,^13,14^ while polymer electrets and ferroelectric polymers deliver long retention and mechanical flexibility in organic memory architectures.^15^ Most recently, sulphur-rich polymers prepared by inverse vulcanisation have enabled probe-based mechanical data storage with repeated write–read–erase cycles and multi-level encoding at room temperature.^16^ Collectively, these advances establish polymers as a robust and versatile class of functional materials for data storage. The PNIPAM-based optical chemical memory introduced here extends this paradigm by exploiting a light-driven, reversible chemical transformation to encode and retain information in interfacial reactivity, rather than electrical conductivity or morphology.

Finally, array-level optical addressing can be engineered photothermally by exploiting wavelength and polarisation: the absorption (and thus heat generation) of anisotropic nanoantennas depends on both, and temperature/heat-source distributions in complex arrays can be designed and mapped under far-field illumination.^1,2,17,18^ However, these capabilities have not previously been used to realise long-lived, reversible chemical state changes on surfaces under uniform illumination.

We therefore introduce an optically programmable chemical memory: a nanostructured surface that can be written into a collapsed polymer state by resonance-matched illumination, store this state metastably over days, and erase it as the polymer rehydrates to its initial configuration. By combining wavelength–polarisation addressing with a thermoresponsive SAM, we demonstrate array-level selectivity, durable storage of chemical information, and complete erasure—providing a general route to erasable, multiplexed control of nanoscale reactivity.

## Nanostructures

**Fig. 1:**
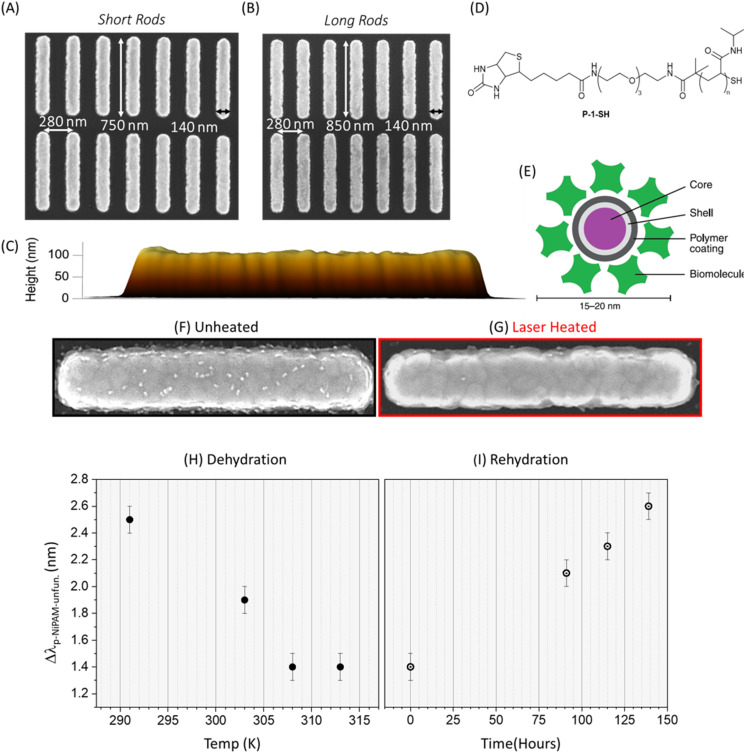
SEM of (A) 750 nm short nanorods array and (B) 850 nm long rods array. (C) AFM image of a rod from the side. (D) Thermal responsive polymer PNIPAM (P-1-SH) molecule (*n* ≈ 76) and (E) schematic of a QD streptavidin conjugate. SEM of (F) unilluminated rod after QDs functionalization and (G) after a 660 nm pulsed laser heating longitudinal polarization at a fluence of 255 mJ cm^−2^. (H) Dehydration and collapse of the PNIPAM SAM is monitored by measuring Δ*λ*_PNiPAM-unfun._ as a function of the temperature of the surrounding buffer solution. (I) Rehydration monitored by measuring Δ*λ*_PNIPAM-unfun._ as a function of time.

To explore aspect-ratio-selective functionalization, we fabricated 1 × 1 mm^2^ arrays of plasmonic gold nanorods with two distinct aspect ratios by electron-beam lithography. Each array contained either “short” rods (∼750 nm long) or “long” rods (∼850 nm long), both with a transverse width of ≈140 nm and a height of ≈110 nm, arranged on a silicon substrate at a 280 nm pitch. The lateral dimensions were confirmed by scanning electron microscopy (SEM), and the heights by atomic force microscopy (AFM) (Fig. 1A–C).

We characterized the optical response of the unfunctionalized nanorods in buffer using reflectance spectroscopy. Spectra were recorded with the polarization aligned along the long axis (0°) and the short axis (90°) of the rods; data for the short and long arrays (SI). In the 0° spectra, enhanced reflectance peaks appear at ∼680 nm for short rods and ∼720 nm for long rods. By contrast, the 90° spectra of both arrays are nearly identical—which is expected, since the rods share the same width—and exhibit a resonance of increased transmission at ∼700 nm. The line shape of the reflectance spectra can be replicated using electromagnetic (EM) numerical simulation (Comsol Multiphysics) (SI).

## Surface functionalisation

A self-assembled monolayer of thiol-terminated, biotin-functionalized poly(*N*-isopropylacrylamide), Fig. 1D, was formed on gold nanorods by immersion in polymer solution for 24 h, a treatment known to produce saturated, well-ordered monolayers within a day on Au surfaces.^19^ Unlike surface-initiated polymer brushes (grafting-from),^20^ which grow high-density chains in an extended conformation, this solution-phase adsorption (grafting-to) deposits preformed chains that maintain their solution-phase coil/helix structure at the interface. Consequently, the biotin head group is accessible to bind to streptavidin functionalised quantum dots (CD), Fig. 1E. Successful SAM formation was confirmed by a 2–3 nm red shift in the plasmon resonance and an ≈10 nm increase in its linewidth (SI). The red shift arises from the higher local refractive index at the metal interface introduced by the SAM,^21^ while the newly formed metal-chemisorbed adsorbate boundary enables chemical interface damping—additional nonradiative decay pathways that broaden the plasmon linewidth,^22^ (Chemical Interface Damping of Surface Plasmon Resonances).

## Thermal properties of SAM

The thermally responsive properties of PNIPAM in solution are well established. It exhibits a lower critical solution temperature (LCST) of ∼316,^4^ above which the polymer undergoes a reversible conformational collapse from an extended helical state to a compact globular form. The polymer used to form the SAM conformed to this behaviour as illustrated with optical transmission and dynamic light scattering measurements, (SI). Below its LCST, PNIPAM chains remain fully hydrated in a random-coil (helical) conformation and the solution is optically clear, transmitting nearly 100% of incident visible light. As the temperature exceeds the LCST, the polymer collapses into dense, hydrophobic globules that form large aggregates and scatter light strongly, producing a sharp, drop in transmittance and increase in the hydrodynamic radius at the transition temperature.

Thermal behaviour of the PNIPAM SAM was evaluated by immersing substrates in buffer at each target temperature for 15 minutes and recording reflectance spectra. After cooling to room temperature, spectra were acquired within 30 minutes. The resulting plasmonic resonance shift (Δ*λ*), relative to an unmodified array, was plotted *versus* temperature, Fig. 1H. Heating to 308 K induced a 1.1 ± 0.2 nm decrease in Δ*λ*, which persisted up to 313 K. To test reversibility, samples were returned to buffer at room temperature for up to 138 hours, with spectra collected periodically, Fig. 1I. Over this period, Δ*λ* gradually recovered, reaching its original value after 138 hours.

To assess the robustness and reproducibility of the PNIPAM-SAM response, additional heating cycles were performed on the same substrate used for the dehydration/rehydration experiment described above, following storage in buffer. The first post-storage heating cycle, conducted 30 days after deposition, produced a plasmonic blue shift of −1.1 nm, within experimental error of the value observed immediately after deposition, confirming that the SAM retained its thermoresponsive behaviour after extended storage. Two further heating cycles were applied on days 31 and 32, before full rehydration of the polymer layer had occurred. The corresponding Δ*λ* values of 0.0 nm and −0.5 nm indicate that the layer remained largely in a collapsed or partially collapsed configuration, showing minimal structural change. After a further week in buffer, a heating cycle on day 39 again produced a blue shift of −1.0 nm, demonstrating complete recovery of the thermoresponsive behaviour (Table 1). These results confirm that the polymer–plasmonic interface is stable and that repeated heating, even under incomplete rehydration, does not degrade the optical or chemical functionality of the surface. This behaviour—slow, diffusion-limited rehydration of a kinetically trapped, partially collapsed configuration—underpins the long-lived written state that defines the optical chemical memory.

**Table 1:** Δ*λ* values recorded after successive heating cycles on the same PNIPAM-SAM substrate

Days post deposition	Heating cycles	Δ*λ*/nm
1	1	−1.1 ± 0.2
30	2	−1.1 ± 0.2
31	3	0.0 ± 0.2
32	4	−0.5 ± 0.2
39	5	−1.0 ± 0.2

Because the temperature at which Δ*λ* decreases closely matches the expected LCST of PNIPAM, we attribute this shift to polymer collapse into a globular conformation. However, our results differ notably from earlier reflectance-based studies of PNIPAM-functionalized gold plasmonic nanoparticles: in those studies, collapse led to a red shift—indicative of an increased effective refractive index in the near field,^8,23^ whereas here we observe a blue shift, signalling a reduced effective index. We believe this can be reconciled by noting that the nanorods studied here are significantly larger than the previously examined nanoparticles (<100 nm). This larger size strongly modifies the EM near field: small nanoparticles (<100 nm) support fields that decay within ∼10 nm of the surface, but larger structures resonating at longer wavelengths generate near fields that extend significantly farther (validated by numerical simulations; SI). Thus, for nanorods, the near field extends beyond the collapsed polymer layer, so collapse replaces higher-index polymer with lower-index aqueous buffer, lowering the average refractive index in the near-field volume and producing a small blue shift. In contrast, for small nanoparticles the near field remains within the collapsed globule, which contains less water and thus increases the local refractive index, yielding a red shift.

The kinetics of collapse/rehydration of PNIPAM SAMs have not been reported. In contrast, PNIPAM-coated gold nanoparticles recover from collapse in ∼100 ns following laser flash photolysis experiments.^8^ Densely grafted PNIPAM brushes on planar substrates reswell fully within seconds to minutes: QCM-D studies show complete rehydration in 1–10 s,^6^ and interferometric measurements confirm recovery in ∼100–200 s.^7^ We attribute the multi-day equilibration of SAMs to several factors: (i) low grafting density (<1 chain per nm^2^) inherent to thiol-gold monolayers, which cause chains to collapse into compact, substrate-pinned globules with minimal free volume;^20^ (ii) effective thiolate-gold anchoring, which prevents chain desorption and limits mobility;^24^ and (iii) enhanced lateral van der Waals interactions in low-density SAMs, which raise the energy barrier to chain re-extension by orders of magnitude compared to brushes.^25^ Together, these features convert an inherently fast LCST-driven transition into a diffusion-limited, kinetically trapped state that only returns to equilibrium over days.

Although the grafting density of the thiol-anchored PNIPAM SAM was not directly measured, literature reports for self-assembled alkanethiol monolayers on Au(111) indicate a close-packed (√3 × √3)*R*30° structure with an area of approximately 21.6 Å^2^ per molecule, corresponding to ≈4.6 molecules per nm^2^.^24^ In contrast, surface-initiated polymer brush architectures typically exhibit grafting densities of ≈0.3–0.5 chains per nm^2^.^26,27^ The PNIPAM SAM formed here by a graft-to process of pre-synthesised, thiol-terminated chains is therefore expected to occupy an intermediate regime—less dense than a small-molecule SAM but more compact than a polymer brush—corresponding to an effective density of <1 chain per nm^2^. While no previous study has reported multi-day rehydration for such low-density thiol-polymer SAMs, the dependence of swelling and collapse kinetics on grafting density is well established: PNIPAM brushes display markedly slower reswelling as chain density decreases.^25^ The slow recovery observed here is therefore consistent with diffusion-limited rehydration in a sparsely grafted, substrate-pinned monolayer.

## Laser driven process

Thermoplasmonic activation of the polymer layer was achieved using a nanosecond pulsed laser (5 ns pulse width, 20 mW average power, 255 mJ cm^−2^ fluence), directed at normal incidence through a 10× objective to a ∼1 mm diameter spot. Nanosecond-pulsed excitation was selected to achieve transient, spatially confined heating of the plasmonic nanorods while minimising unwanted global temperature rise. Each pulse drives a rapid local temperature increase at the illuminated nanostructures, sufficient to exceed the PNIPAM lower critical solution temperature (LCST) at the metal–solution interface, followed by rapid cooling between pulses. Because the duty cycle is low, the time-averaged thermal load on the surrounding medium remains small, suppressing bulk heating and preserving the reversibility of the polymer layer. This confinement enables wavelength- and polarisation-selective switching of individual nanorod arrays without cross-talk. In contrast, continuous-wave illumination at comparable average power would produce lower peak temperatures and broader, quasi-steady-state thermal profiles, reducing spatial selectivity of the polymer collapse.

Similar spatiotemporal confinement of pulsed thermoplasmonic heating—where high instantaneous temperatures are achieved locally without raising the bulk medium above equilibrium—has been reported previously for metal nanoparticle systems.^2,28^ The pulsed-excitation regime therefore provides the required combination of high local peak temperature and low average heating to induce site-specific PNIPAM collapse while avoiding nonspecific activation or damage.

By tuning the laser wavelength and polarization, we selectively excited the plasmonic resonances of nanorod arrays with specific dimensions, producing localized heating through nonradiative decay.^2^ Arrays were exposed for 2 minutes to various combinations of wavelength (660–1000 nm) and polarization (longitudinal or transverse relative to the nanorod long axis). Although arrays were typically exposed for 2 min to ensure complete activation, this duration represents a conservative upper limit rather than a kinetic requirement. The nanosecond laser operated at 20 Hz, so each exposure comprised approximately 2400 intense 5 ns pulses. Each pulse produces a rapid, localized temperature spike that transiently raises the polymer–metal interface above the PNIPAM LCST. In nanoparticle systems, collapse is initiated within the nanosecond pulse and reswelling occurs in <100 ns,^8^ while local thermal equilibration occurs on sub-microsecond scales in plasmonic structures.^28^ Consequently, only a few seconds of irradiation at 20 Hz are expected to establish a stable written state, and the 2 min exposure was chosen to guarantee uniform switching across the illuminated region rather than to reflect the intrinsic write-speed limit.

To probe the functional state of the polymer layer after laser exposure, the samples were incubated with streptavidin-functionalized quantum dots. These bind only to exposed biotin groups, providing a direct visual readout of surface chemical activity.^29^ SEM imaging revealed substantial differences in quantum dot binding between irradiated and unexposed arrays. As shown in Fig. 1F and G, unheated nanorods exhibited dense QD coverage, whereas irradiated rods displayed significantly reduced binding, consistent with thermally induced polymer collapse and biotin deactivation. The contrast in QD binding underpins the form-factor-selective functionalization strategy explored in the following sections.

The thermally induced switching of the PNIPAM layer was first monitored *via* reflectance spectroscopy (SI), exploiting the sensitivity of the nanorod plasmonic resonance to changes in the surrounding dielectric environment.^30,31^ For uncoated arrays in water, the longitudinal (0°) reflectance spectra revealed resonance peaks centred at ∼680 nm for short nanorods and ∼720 nm for long nanorods. Following adsorption of the PNIPAM monolayer, a red shift of 2–3 nm was observed for both structures.^32^

**Fig. 2:**
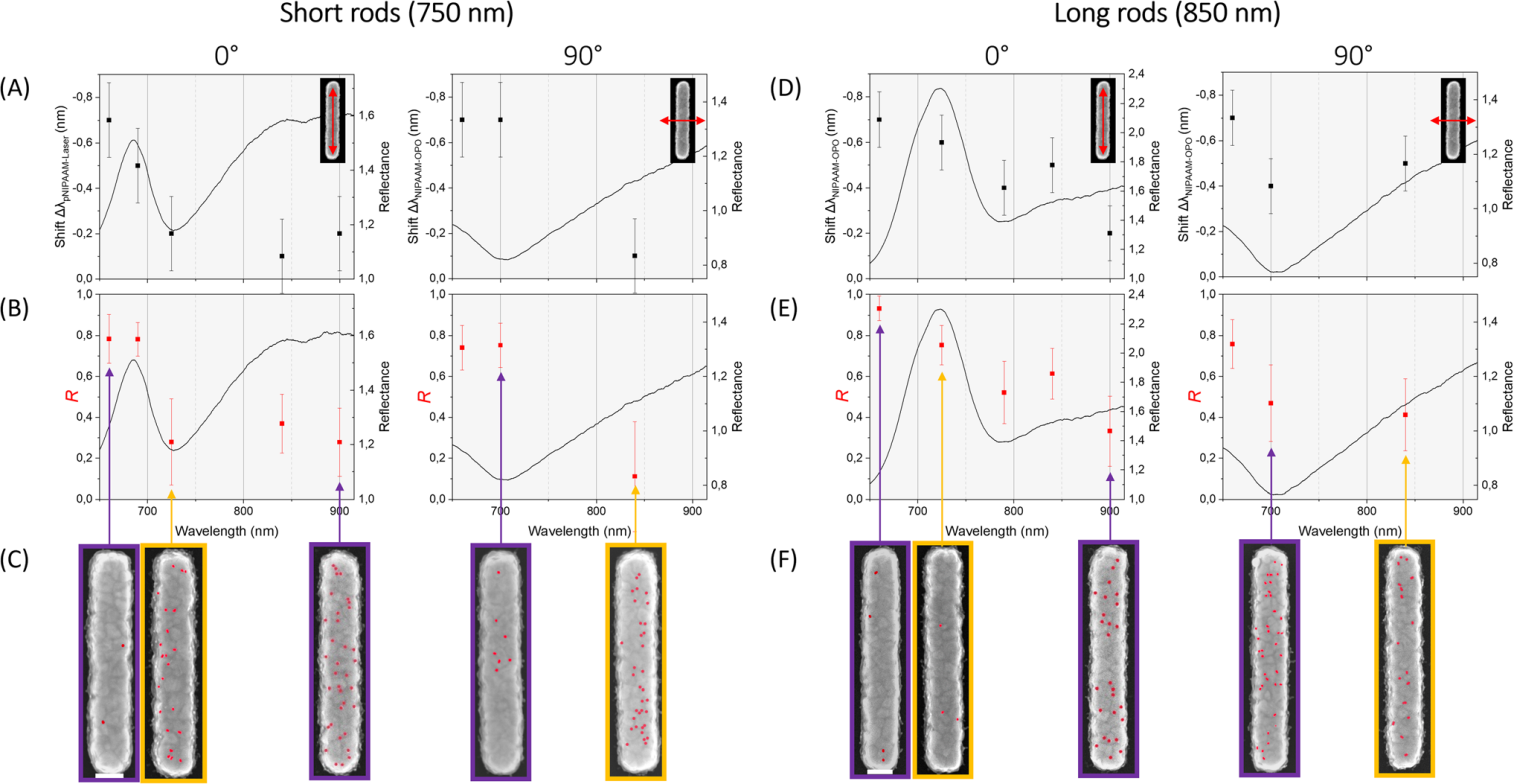
Short- (A–C) and long-rod (D–F) data under pulsed laser excitation. (A and D) Wavelength-dependent reflectance shift (Δ*λ*__(illum–unillum)_) for longitudinal (0°, left panels) and transverse (90°, right panels) polarization; red arrows indicate the rod orientation relative to the incident field. Experimental reflectance spectra for each polarization are overlaid. Error bars denote the standard deviation of five spectra. (B and E) Reduction factor *R* (red) as a function of excitation wavelength for both polarizations. Error bars correspond to the standard deviation of QD counts across 30 rods. (C and F) Representative SEM images showing QD binding (false-coloured red) under irradiation conditions marked by the purple and yellow arrows in (A and D). Scale bar = 100 nm.

After laser irradiation, a wavelength-dependent blue shift was observed in the plasmonic resonance peak position, indicating a local reduction in refractive index consistent with the thermally induced collapse of the PNIPAM layer.^33^ The magnitude of the shift varied with both laser wavelength and polarization, with more pronounced shifts occurring when the laser excitation was close to the resonance of the nanorod array. This behaviour is summarised in Fig. 2A and D, which plot the change in resonance wavelength (Δ*λ*) as a function of laser wavelength for short and long nanorods, respectively.

To directly assess the chemical functionality of the polymer-coated surfaces, we quantified the number of streptavidin-conjugated quantum dots bound to each nanorod using SEM imaging (SI). A reduction factor, *R*, was defined as the ratio of the average number of QDs bound to irradiated rods compared to unirradiated controls. An *R* value of 1 indicates complete suppression of binding (*i.e.*, full polymer collapse), while *R* ≈ 0 signifies no change from unheated arrays. The variation of *R* with laser parameters is shown in Fig. 2B and E.

This analysis revealed a strong dependence of QD binding on both nanorod geometry and laser excitation conditions. For example, under 725 nm longitudinal (0°) illumination, long rods showed a marked reduction in QD coverage (*R* ≈ 0.9), whereas short rods remained largely functional (*R* ≈ 0.1). The inverse behaviour was observed at 700 nm with transverse (90°) polarization, where QD binding was suppressed on short rods but retained on long rods. Fig. 2C and F present representative SEM images of the corresponding QD binding patterns under these irradiation conditions, providing direct visual confirmation of the quantitative trends shown in Fig. 2A–E.

These results confirm that by tuning the excitation wavelength and polarization, it is possible to selectively activate or suppress surface chemistry based on nanostructure form factor. Crucially, the trends in plasmonic blue shift (Δ*λ*) and QD reduction factor (*R*) exhibit strong correlation: both reflect the same underlying resonance-dependent heating mechanism. This is clearly evidenced by the agreement between short (Fig. 2A and B) and long rods (Fig. 2D and E), where maxima in Δ*λ* align closely with the conditions that yield maximum suppression of QD binding. These correspondences validate the use of far-field optical measurements as a quantitative and predictive proxy for the functional state of surface chemistry.

The thermoresponsive behaviour of the PNIPAM layer was found to be gradual and temperature-dependent, consistent with previous studies on polymer collapse dynamics.^8,34^ As a result, partial switching and intermediate QD densities were observed under some irradiation conditions, enabling graded control over surface reactivity. These findings underscore the tunability of the system and demonstrate that far-field optical inputs can programmably modulate chemical functionality at the level of individual nanostructure arrays across macroscopic areas.

**Fig. 3:**
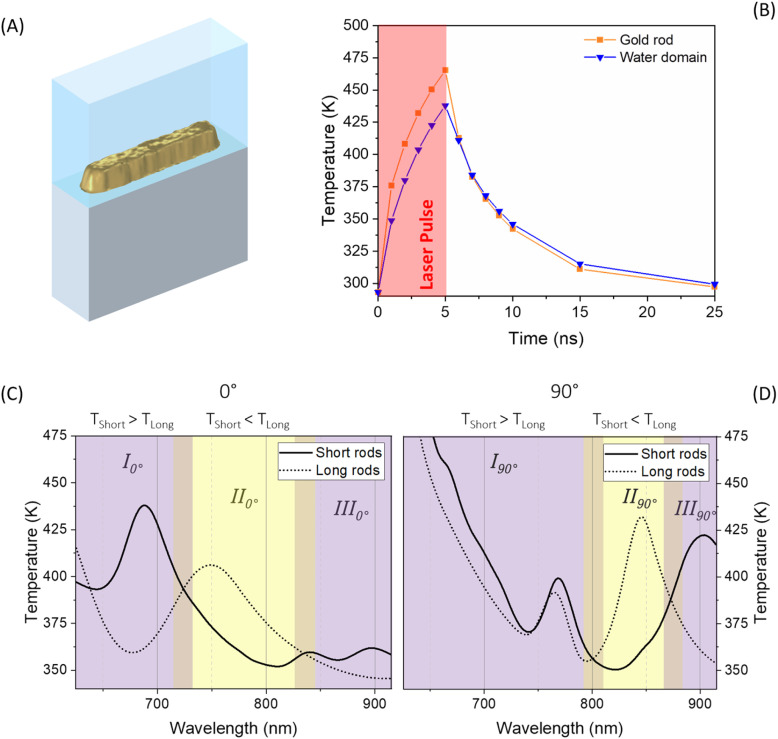
(A) COMSOL model of a short gold nanorod derived from AFM topography. (B) Temporal evolution of the average temperature in the nanorod (orange) and in a 10 nm water domain adjacent to its surface (blue) under a 5 ns laser pulse at *λ* = 690 nm with longitudinal (0°) polarization. (C and D) Simulated peak temperatures in a 10 nm water domain surrounding short rods (solid lines) and long rods (dotted lines) as a function of excitation wavelength for (C) longitudinal (0°) and (D) transverse (90°) polarization. The purple regions mark wavelength ranges where short rods heat more strongly than long rods, while yellow regions indicate the opposite trend.

To understand the mechanism driving the observed selective switching behaviour, we conducted finite-element simulations of electromagnetic absorption and transient heat transfer using COMSOL Multiphysics. The model geometry was constructed from AFM topographic data of the fabricated nanorods, rather than idealised analytical shapes. This realistic geometry captures fabrication-related features such as sidewall tapering, curvature, and edge asymmetries, enabling more accurate prediction of both plasmonic absorption and heat localisation. The COMSOL simulation geometry is shown in Fig. 3A, and represents a methodological refinement over conventional simulations based on idealised structures.

The thermal response of the system was calculated using the transient heat diffusion equation:1
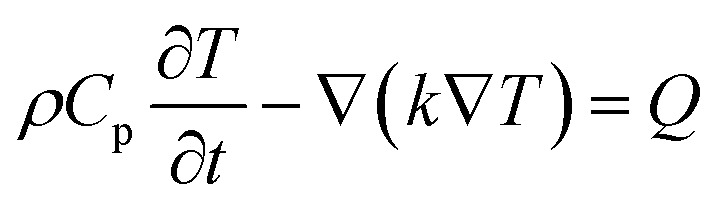
where *ρ* is the density, *C*_p_ the specific heat, *k* the thermal conductivity, *T* the temperature, and *Q* the electromagnetic heat source term (power density). This equation (eqn (1)) was solved using time-dependent conditions matching the experimental laser pulse duration (5 ns).

The power density *Q* was derived from the simulated electric field distributions using:2
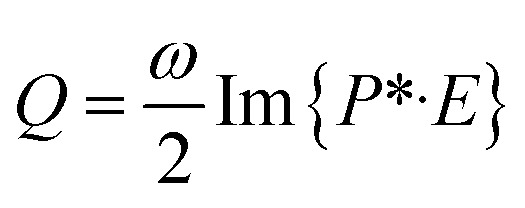
where *E* is the electric field and *P* the polarisation field. In isotropic media, this simplifies to:3
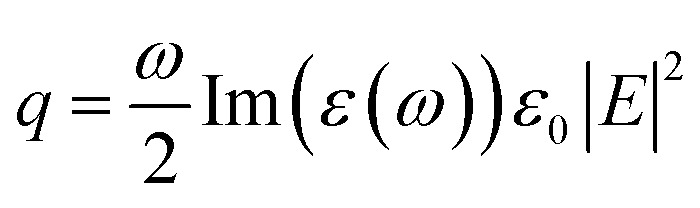
Eqn (2) and (3) were used to quantify the absorption profile within the nanorods, with optical constants taken from Johnson and Christy.^35^

The temporal evolution of temperature during a single 5 ns laser pulse is shown in Fig. 3B. The gold nanostructure reaches its peak temperature rapidly during the pulse, while the surrounding water heats more slowly and to a lower maximum. This transient mismatch indicates that the PNIPAM layer at the rod–solution interface experiences a brief thermal spike sufficient to trigger collapse, while the bulk remains below the LCST (∼316 K).^4^

The simulated peak temperatures for short and long rods under varying excitation conditions are summarised in Fig. 3C and D, which identifies three thermal regimes (Regions I–III, subscripts label 0°/90°) based on the relative heating of the two geometries. Notably, the simulations show that peak temperatures exceed the LCST of PNIPAM across all wavelengths and polarisation conditions. However, switching is only observed experimentally under resonance conditions—and under these conditions, complete switching is achieved, as evidenced by the full suppression of quantum dot binding (*R* ≈ 1). In both Region I and Region III, the short rods are hotter than the long rods, while in Region II, the long rods are hotter than the short rods. This inversion enables selective switching of the long rods. Region II spans different wavelength ranges for 0° and 90° polarisation, meaning that polarisation can be used to selectively target nanorod geometries for functionalisation at a fixed wavelength. This provides a mechanistic explanation for the polarisation-dependent switching observed experimentally (Fig. 2).

This apparent paradox—that switching is selective despite all simulated conditions exceeding the LCST—can be resolved by considering the non-equilibrium nature of pulsed laser heating. The LCST defines a thermodynamic transition temperature under slow, uniform heating,^4^ but in our system, thermal excitation occurs over nanosecond timescales and is followed by rapid cooling. Under these conditions, polymer collapse requires not only exceeding the LCST but also sufficient residence time above it for dehydration and conformational reorganisation. Off-resonance conditions may briefly exceed 316 K, but the combination of shorter duration and smaller effective volume reduces the likelihood of collapse. This kinetic explanation is consistent with previous reports of rate-dependent behaviour in PNIPAM films.^8^ To further probe the threshold conditions for switching, this experiment was replicated at half the laser power (10 mW average; see SI). The results showed that QD binding was only marginally reduced for the combinations of 690 nm/0° on short rods (*R* = 0.2 ± 0.15), and 660 nm/90° on both short and long rods (*R* = 0.3 ± 0.17 and *R* = 0.26 ± 0.2 respectively). This indicates that only these resonance-matched conditions raised the PNIPAM layer above its LCST, even under reduced power, further confirming the selectivity of resonant nanoheating.

**Fig. 4:**
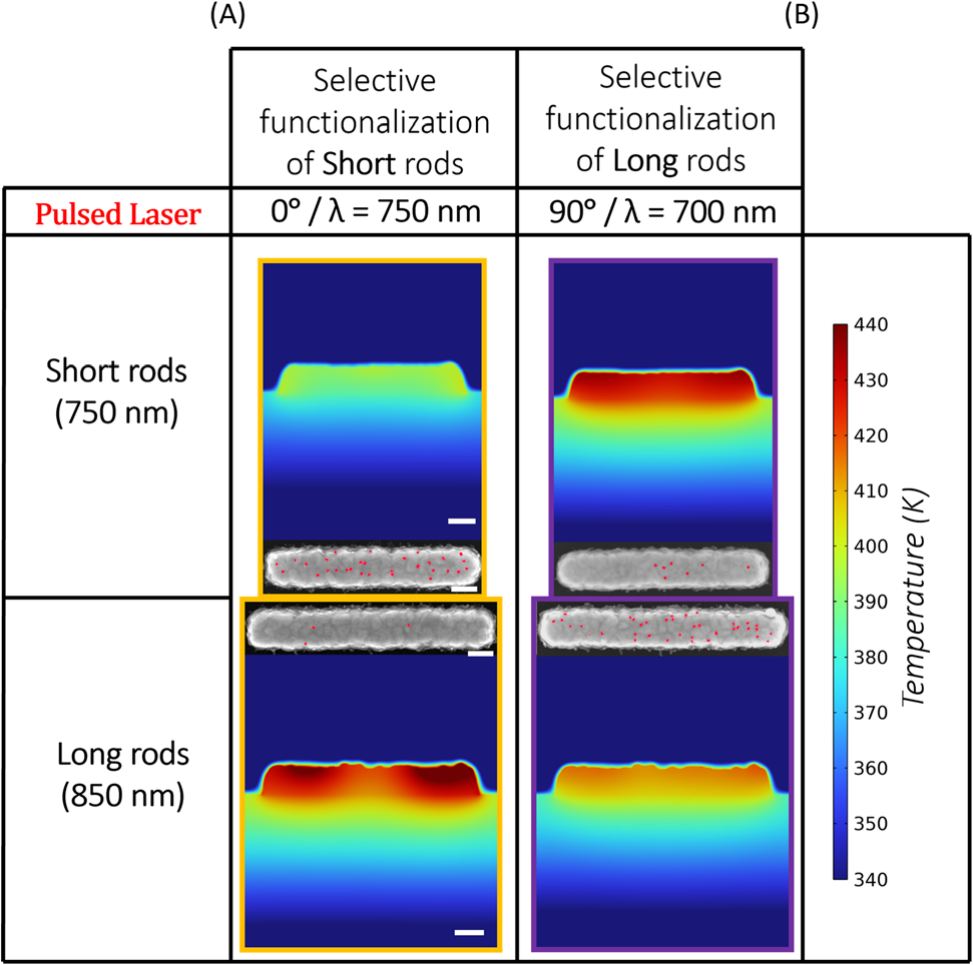
Simulated heat maps (top) and corresponding SEM images (bottom) of short and long nanorods under pulsed laser illumination. (A) Selective QD functionalization observed under conditions where long rods heat more strongly (yellow zone: *λ* = 725 nm, 0° polarization). (B) Selective QD functionalization observed under conditions where short rods heat more strongly (purple zone: *λ* = 700 nm, 90° polarization). In SEM images, QDs are false-coloured red; scale bar = 100 nm.

The spatial temperature distribution at the end of the pulse is shown in Fig. 4A and B, which confirms that heating is strongly confined to the nanostructure and its immediate surroundings. Most of the heat dissipates vertically into the silicon substrate, with minimal lateral diffusion into the surrounding fluid. This spatial confinement explains the lack of thermal cross-talk between adjacent structures and supports the interpretation of form-factor-specific switching under far-field illumination.

Together, these simulations provide a quantitative, time-resolved framework for interpreting the experimental results. The use of realistic nanostructure geometry, transient thermal modelling, and polarisation-resolved absorption analysis confirms that resonance-enhanced, structure-specific nanoheating enables programmable switching of surface chemistry using only optical input parameters.

We have shown that plasmonic nanoarrays coated with a thermoresponsive polymer SAM can act as an optically programmable chemical memory, with interfacial functionality that can be written, stored, and erased under far-field illumination. The central advance lies not in triggering collapse of PNIPAM, which has long been possible, but in achieving a metastable state that endures for more than a day, orders of magnitude longer than the transient responses reported for brushes or core–shell nanoparticles. This finding demonstrates that the kinetics of polymer collapse can be re-engineered at the monolayer level to produce durable, reversible chemical states.

The magnitude of the plasmonic wavelength shift (≈1–2 nm) observed upon polymer collapse is reproducible and comparable to values commonly used in plasmonic sensing. Nevertheless, such small spectral changes would provide limited contrast for a practical memory device. The present work should therefore be regarded as a proof-of-concept demonstration of an optically programmable chemical state rather than an optimised data-storage architecture.

A promising strategy to achieve greater optical contrast would be to employ chiral plasmonic nanostructures functionalised with PNIPAM. In these systems, the collapse of the polymer would significantly modify the local chiral electromagnetic field, thereby altering the optical rotation or circular dichroism of transmitted or reflected light. By placing the structure between a pair of linear polarisers oriented near an extinction angle, one could realise a near-zero transmission (OFF) state before polymer collapse and a strong transmission (ON) state after collapse, providing a robust binary optical output.

Jack *et al.*^36^ have already shown that thermoresponsive polymer-coated chiral nanostructures exhibit a structural transformation above a critical temperature, accompanied by a change in optical rotation of up to 2.5° at a fixed wavelength. Such a change is readily measurable and demonstrates that polymer-induced modulation of chiral optical response can deliver high-contrast optical switching. These findings support the feasibility of implementing a chiral PNIPAM-based read-out scheme capable of producing binary ON/OFF states with far higher optical contrast than achievable from small plasmonic wavelength shifts alone.

The results presented here demonstrate the core functional characteristics of a chemical memory at the nanoscale. The multiple SEM images from which the data in Fig. 2 were obtained, together with additional examples in the SI, show a consistent reduction in quantum-dot binding across many nanostructures within the irradiated region. This reproducible binary contrast between written (collapsed) and erased (hydrated) states confirms that the optical and chemical responses are spatially uniform. Repeated reflectance measurements on the same regions produced identical spectra, showing that optical readout is non-destructive. The thermal-cycling data verify that PNIPAM collapse and recovery are reversible.

These findings establish a proof-of-concept for an optically programmable chemical memory that exhibits stable written states, reliable readout, and reversible erasure. Quantitative metrics such as long-term endurance, bit-error rate, and large-area uniformity lie beyond the scope of this first demonstration but will be the focus of future work aimed at developing a fully characterised optical chemical-memory architecture.

Beyond establishing the principle of chemical memory, our results reveal that geometry-selective thermoplasmonic heating provides a practical means of programming surface chemistry across co-fabricated arrays without masks or serial processing. This capability suggests new routes to multiplexed biosensing, where binding events can be locally gated in space and time, and to programmable nanofabrication, where reactivity can be written and erased optically. More broadly, integrating long-lived polymer conformational switching with photonic architectures points to a general strategy for designing erasable and reconfigurable chemical functionality at the nanoscale.

## Author contributions

The manuscript was written through contributions of all authors. All authors have given approval to the final version of the manuscript.

## Conflicts of interest

There are no conflicts to declare.

## Supplementary Material

SC-OLF-D5SC07368E-s001

## Data Availability

The datasets supporting this article are available in the supplementary information (SI). Additional data are available from the corresponding author on reasonable request. Supplementary information: experimental details and methods, numerical simulations and details, SEM data. See DOI: https://doi.org/10.1039/d5sc07368e.

## References

[cit1] Baffou G., Quidant R., García de Abajo F. J. (2010). Nanoscale Control of Optical Heating in Complex Plasmonic Systems. ACS Nano.

[cit2] Baffou G., Quidant R. (2013). Thermo-plasmonics: using metallic nanostructures as nano-sources of heat. Laser Photon. Rev..

[cit3] Ou W., Zhou B., Shen J., Zhao C., Li Y. Y., Lu J. (2021). Plasmonic metal nanostructures: concepts, challenges and opportunities in photo-mediated chemical transformations. iScience.

[cit4] Schild H. G. (1992). poly (n-isopropylacrylamide) - experiment, theory and application. Prog. Polym. Sci..

[cit5] Wurm F. R., Boyer C., Sumerlin B. S. (2021). Progress on Stimuli-Responsive Polymers. Macromol. Rapid Commun..

[cit6] Ishida N., Biggs S. (2007). Direct Observation of the Phase Transition for a Poly(N-isopropylacryamide) Layer Grafted onto a Solid Surface by AFM and QCM-D. Langmuir.

[cit7] van Duinen D., Butt H.-J., Berger R. (2019). Two-Stage Collapse of PNIPAM Brushes: Viscoelastic Changes Revealed by an Interferometric Laser Technique. Langmuir.

[cit8] Murphy S., Jaber S., Ritchie C., Karg M., Mulvaney P. (2016). Laser Flash Photolysis of Au-PNIPAM Core–Shell Nanoparticles: Dynamics of the Shell Response. Langmuir.

[cit9] Hukum K. O., Caykara T., Demirel G. (2023). Thermoresponsive Polymer Brush-Decorated 3-D Plasmonic Gold Nanorod Arrays as an Effective Plasmonic Sensing Platform. ACS Appl. Polym. Mater..

[cit10] Albers W. M., Auer S., Helle H., Munter T., Vikholm-Lundin I. (2009). Functional characterisation of Fab '-fragments self-assembled onto hydrophilic gold surfaces. Colloids Surf., B.

[cit11] Prabhathan P., Sreekanth K. V., Teng J., Ko J. H., Yoo Y. J., Jeong H.-H., Lee Y., Zhang S., Cao T., Popescu C.-C. (2023). *et al.*, Roadmap for phase change materials in photonics and beyond. iScience.

[cit12] Zhou W., Farmakidis N., Feldmann J., Li X., Tan J., He Y., Wright C. D., Pernice W. H. P., Bhaskaran H. (2022). Phase-change materials for energy-efficient photonic memory and computing. MRS Bull..

[cit13] Lin W.-P., Liu S.-J., Gong T., Zhao Q., Huang W. (2014). Polymer-Based Resistive Memory Materials and Devices. Adv. Mater..

[cit14] Lee S., Kim S., Yoo H. (2021). Contribution of Polymers to Electronic Memory Devices and Applications. Polymers.

[cit15] Eng Y. J., Weng Y.-H., Oh A. B., Liu C.-L., Chan J. M. W. (2024). Polymer electrets for organic nonvolatile memory devices: Recent advances. Mater. Today Chem..

[cit16] Mann A. K., Tonkin S. J., Sharma P., Gibson C. T., Chalker J. M. (2025). Probe-Based Mechanical Data Storage on Polymers Made by Inverse Vulcanization. Advanced Science.

[cit17] Chen J., Ye Z., Yang F., Yin Y. (2021). Plasmonic Nanostructures for Photothermal Conversion. Small Sci..

[cit18] Petronella F., Madeleine T., De Mei V., Zaccagnini F., Striccoli M., D'Alessandro G., Rumi M., Slagle J., Kaczmarek M., De Sio L. (2023). Thermoplasmonic Controlled Optical Absorber Based on a Liquid Crystal Metasurface. ACS Appl. Mater. Interfaces.

[cit19] Smith J. G., Jain P. K. (2016). Kinetics of self-assembled monolayer formation on individual nanoparticles. Phys. Chem. Chem. Phys..

[cit20] Cho E. C., Kim Y. D., Cho K. (2004). Thermally responsive poly(N-isopropylacrylamide) monolayer on gold: synthesis, surface characterization, and protein interaction/adsorption studies. Polymer.

[cit21] Mayer K. M., Hafner J. H. (2011). Localized Surface Plasmon Resonance Sensors. Chem. Rev..

[cit22] Lee S. A., Link S. (2021). Chemical Interface Damping of Surface Plasmon Resonances. Acc. Chem. Res..

[cit23] Gehan H., Mangeney C., Aubard J., Lévi G., Hohenau A., Krenn J. R., Lacaze E., Félidj N. (2011). Design and Optical Properties of Active Polymer-Coated Plasmonic Nanostructures. J. Phys. Chem. Lett..

[cit24] Love J. C., Estroff L. A., Kriebel J. K., Nuzzo R. G., Whitesides G. M. (2005). Self-Assembled Monolayers of Thiolates on Metals as a Form of Nanotechnology. Chem. Rev..

[cit25] Malham I. B., Bureau L. (2010). Density Effects on Collapse, Compression, and Adhesion of Thermoresponsive Polymer Brushes. Langmuir.

[cit26] Reese C. J., Boyes S. G. (2021). New methods in polymer brush synthesis: Non-vinyl-based semiflexible and rigid-rod polymer brushes. Prog. Polym. Sci..

[cit27] Wang R., Wei Q., Sheng W., Yu B., Zhou F., Li B. (2023). Driving Polymer Brushes from Synthesis to Functioning. Angew. Chem., Int. Ed..

[cit28] Jauffred L., Samadi A., Klingberg H., Bendix P. M., Oddershede L. B. (2019). Plasmonic Heating of Nanostructures. Chem. Rev..

[cit29] Hajji M., Cariello M., Gilroy C., Kartau M., Syme C. D., Karimullah A., Gadegaard N., Malfait A., Woisel P., Cooke G. (2021). *et al.*, Chiral Quantum Metamaterial for Hypersensitive Biomolecule Detection. ACS Nano.

[cit30] Mogensen K. B., Kneipp K. (2014). Size-Dependent Shifts of Plasmon Resonance in Silver Nanoparticle Films Using Controlled Dissolution: Monitoring the Onset of Surface Screening Effects. J. Phys. Chem. C.

[cit31] Anker J. N., Hall W. P., Lyandres O., Shah N. C., Zhao J., Van Duyne R. P. (2008). Biosensing with plasmonic nanosensors. Nat. Mater..

[cit32] Li M., Bresson B., Cousin F., Fretigny C., Tran Y. (2015). Submicrometric Films of Surface-Attached Polymer Network with Temperature-Responsive Properties. Langmuir.

[cit33] Honda M., Saito Y., Smith N. I., Fujita K., Kawata S. (2011). Nanoscale heating of laser irradiated single gold nanoparticles in liquid. Opt. Express.

[cit34] Lee S. G., Pascal T. A., Koh W., Brunello G. F., Goddard III W. A., Jang S. S. (2012). Deswelling Mechanisms of Surface-Grafted Poly(NIPAAm) Brush: Molecular Dynamics Simulation Approach. J. Phys. Chem. C.

[cit35] Johnson P. B., Christy R. W. (1972). Optical Constants of the Noble Metals. Phys. Rev. B.

[cit36] Jack C., Karimullah A. S., Tullius R., Khorashad L. K., Rodier M., Fitzpatrick B., Barron L. D., Gadegaard N., Lapthorn A. J., Rotello V. M. (2016). *et al.*, Spatial control of chemical processes on nanostructures through nano-localized water heating. Nat. Commun..

