# Supplementary material for: Optical write–erase chemical memory state in plasmonic nanoarrays
Source: Chem Sci. 2025 Dec 3;17(5):2694–702. doi: 10.1039/d5sc07368e (PMC12690844; doi:10.1039/d5sc07368e)
Supplement: SC-017-D5SC07368E-s001 [file SC-017-D5SC07368E-s001.pdf]

Supplementary Information (SI) for Chemical Science.
This journal is © The Royal Society of Chemistry 2025

Supporting Information

Optical Write–Erase Chemical Memory in Plasmonic Nanoarrays.

Victor Tabouillot[1], Muhammad Murad[1], Rahul Kumar[1], Paula L. Lalaguna[1], Maryam Hajji[1], Affar Karimullah[1], Nikolaj Gadegaard[2], Aurélie Malfait[3], Patrice Woisel[3], Graeme Cooke[1], and Malcolm Kadodwala[1*]*

[1] School of Chemistry, Joseph Black Building, University of Glasgow, Glasgow, G12 8QQ, UK

[2] School of Engineering, Rankine Building, University of Glasgow, Glasgow G12 8LT, U.K

[3]Univ. Lille, CNRS, INRAE, Centrale Lille, UMR 8207 - UMET - Unité Matériaux et Transformations, F-59000 Lille, France

Nanostructure Fabrication: Gold plasmonic nanorod arrays were fabricated on silicon substrates using electron-beam lithography (EBPG 5200) on a positive PMMA resist. Following development, a 5 nm Ti adhesion layer and 100 nm of gold were deposited via thermal evaporation (Plassys MEB-400s), and lift-off was performed using a bi-layer process. Two distinct nanorod lengths (750 nm and 850 nm) were patterned into separate square arrays (1 mm × 1 mm).

Polymer Functionalisation: Poly(N-isopropylacrylamide) (p-NIPAM) functionalised with biotin-terminated thiol side groups was synthesised as described previously[1]. After oxygen plasma treatment (80 W, 2 min), substrates were immersed in 0.5 g/mL p-NIPAM in PBS buffer (pH 7.4) for 24 h, then rinsed with PBS to remove unbound polymer.

Quantum Dot Labelling: After laser treatment, reflectance spectra were acquired, and samples were incubated overnight in a dark environment with a PBS solution containing streptavidin-functionalised quantum dots (14 µg/mL; Qdot 705, Invitrogen). Unbound QDs were removed by PBS rinsing.

Imaging and Morphology: Atomic force microscopy (AFM; Bruker Dimension Icon) was used to determine nanorod height. Scanning electron microscopy (SEM; Hitachi SU8240) provided lateral dimensions and pitch. QDs were visualised using secondary electron contrast between gold and semiconductor components.

Far field ORD measurements: These were carried out using a polarization microscope. This custom-built instrument can record ORD and reflectivity spectra. The light is generated by a 50W tungsten halogen light source from Thorlab, the beam passes through a nanoparticle polarizer (Thorlab) and then sent to a beam splitter (Thorlab 50:50 700 nm-1000 nm CCM-1BSO14/M). The light is then diverted and its polarization changes by 90°, which goes to the 10× lens with 0.3 numerical aperture Olympus UPlanFLN. The light reflected from the sample

passes through the beam splitter and traverses another calcite polarizer (out-put polarizer). The beam of light is passing through a second beam splitter to reach a camera (Thorlab CMOS camera DCC1645C) and the spectrometer (Ocean Optic USB 4000). Using Thorlab camera software, it is possible to focus on the nanostructures arrays and align the sample to the linearly polarized incident beam (**Figure S1**).

Reflectance measurements*:* These were collected from all the arrays on the sample was recorded at every step of this study using the polarization setup. These results were obtained by dividing the spectrum taken on the nanostructures by the spectrum reflected from the Si wafer background:

$$Reflectance = \frac{(I_{nanostructure})}{(I_{background})} \quad (1)$$

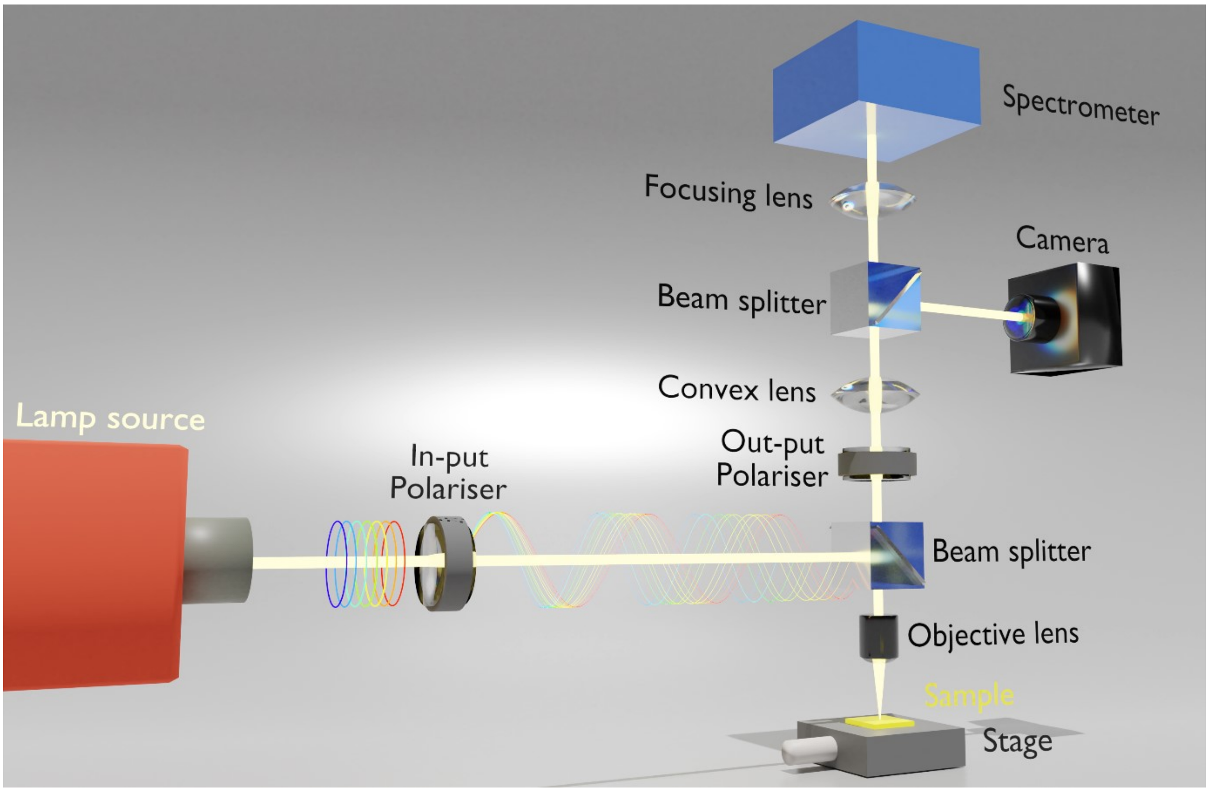

Figure S1. Polarization microscope setup used to measure the reflectance of the sample.

The reflectance spectra and their respective plasmonic resonance peak position at each step of this study are displayed in this section (see **Figure S2**). The curves in blue were taken in water after acetone and O_2 plasma cleaning. Once the p-NIPAAM SAM was deposited, the spectra were measured in PBS and are represented in green. After the pulsed laser heating at different wavelengths and for the 0° and 90° polarization angle, the reflectance spectra were taken and colored red on the plots. Lastly, after the QDs have been deposited and the solution has been rinsed, the reflectance of each array was taken again, and are colored in purple in **Figure S2**.

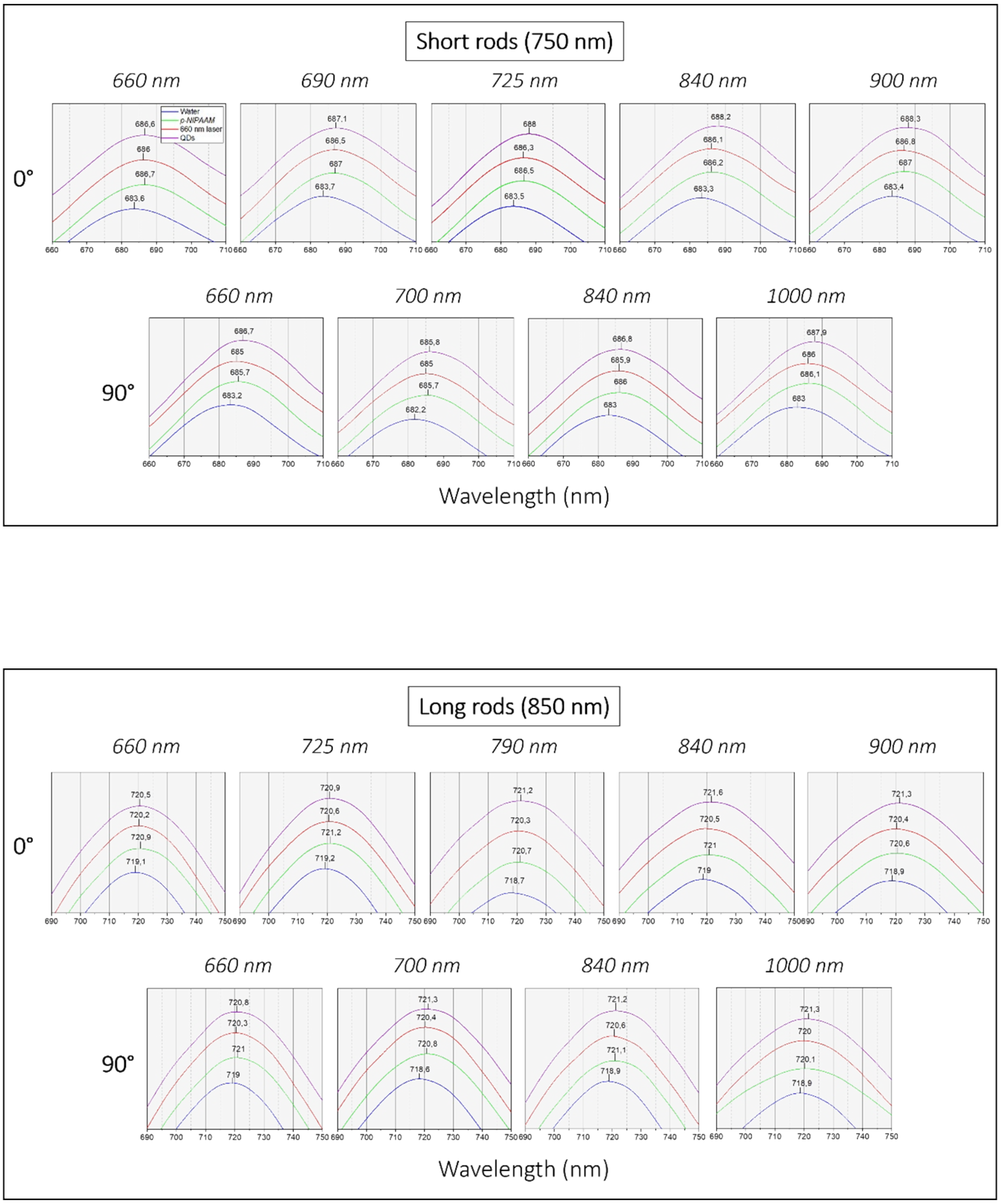

Figure S2. Reflectance spectra of the short rod arrays (top panel) and long rods (bottom panel) taken in water (blue), after *p-NIPAAM* deposition (green), following the laser heating at the wavelength indicated (red) and finally after the QDs functionalization (purple).

Laser nanoheating: Nanostructures were irradiated using a tunable nanosecond pulsed laser (Opotek Radiant SE 532 LD; 5 ns FWHM pulse duration, 20 Hz repetition rate) delivering a 6.5 mm diameter, linearly polarised beam over the range 650–2600 nm. Beam polarisation and power were controlled with a pair of Glan–Laser polarisers, and beam height was adjusted using a Thorlabs RS99/M periscope. The beam was reflected onto the sample at normal incidence via a non-polarising beam splitter and focused through an Olympus UPlanFLN 10×/0.30 NA objective, yielding a ~1 mm diameter spot that matched the array size. Two experimental fluence conditions were used: 255 mJ cm^{-2} (20 mW) and 127 mJ cm^{-2} (10 mW). Arrays were exposed for 2 min at each condition. The excitation wavelength was selected with a ±1 nm tolerance, and polarisations were defined as 0° (along the long axis) or 90° (short axis) by rotating the sample by 90°. Beam alignment and focusing were monitored in situ with a Blackfly USB3 camera. Delivered laser power at the sample plane was verified using a calibrated thermal sensor (Thorlabs S425C). A schematic of the setup is shown in **Figure S3**.

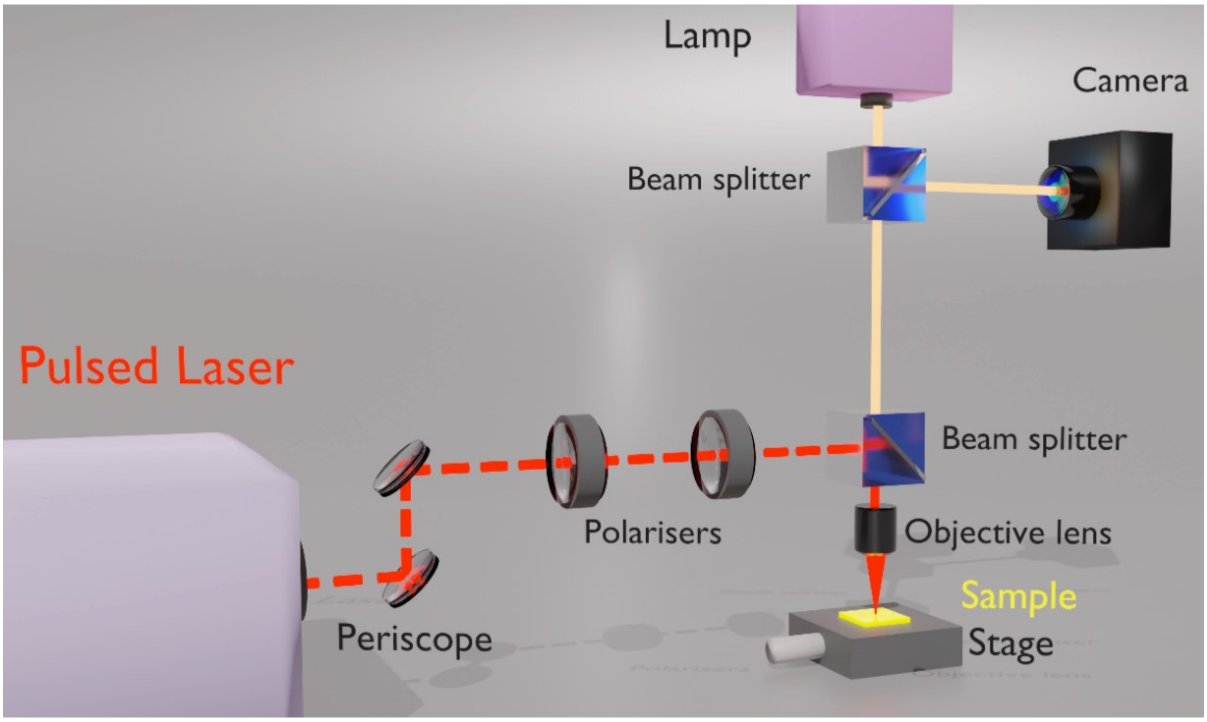

Figure S3. Schematic of the polarized pulsed laser heating setup.

Power dependency*:* Two experiments were conducted using different laser powers: 255 mJ/cm² (20 mW) for one and 127 mJ/cm² (10 mW) for the other. The laser power at each wavelength used in the experiments was measured at the sample position (see **Figure S4** (a) and (b)). This measurement could not be performed with the objective lens in place, as the resulting spot size was too small for the power meter to accurately detect. Instead, a Thorlabs S425C thermal power sensor head, paired with a PM100D compact power meter console, was used. An average of 100 measurements was taken for each setting.

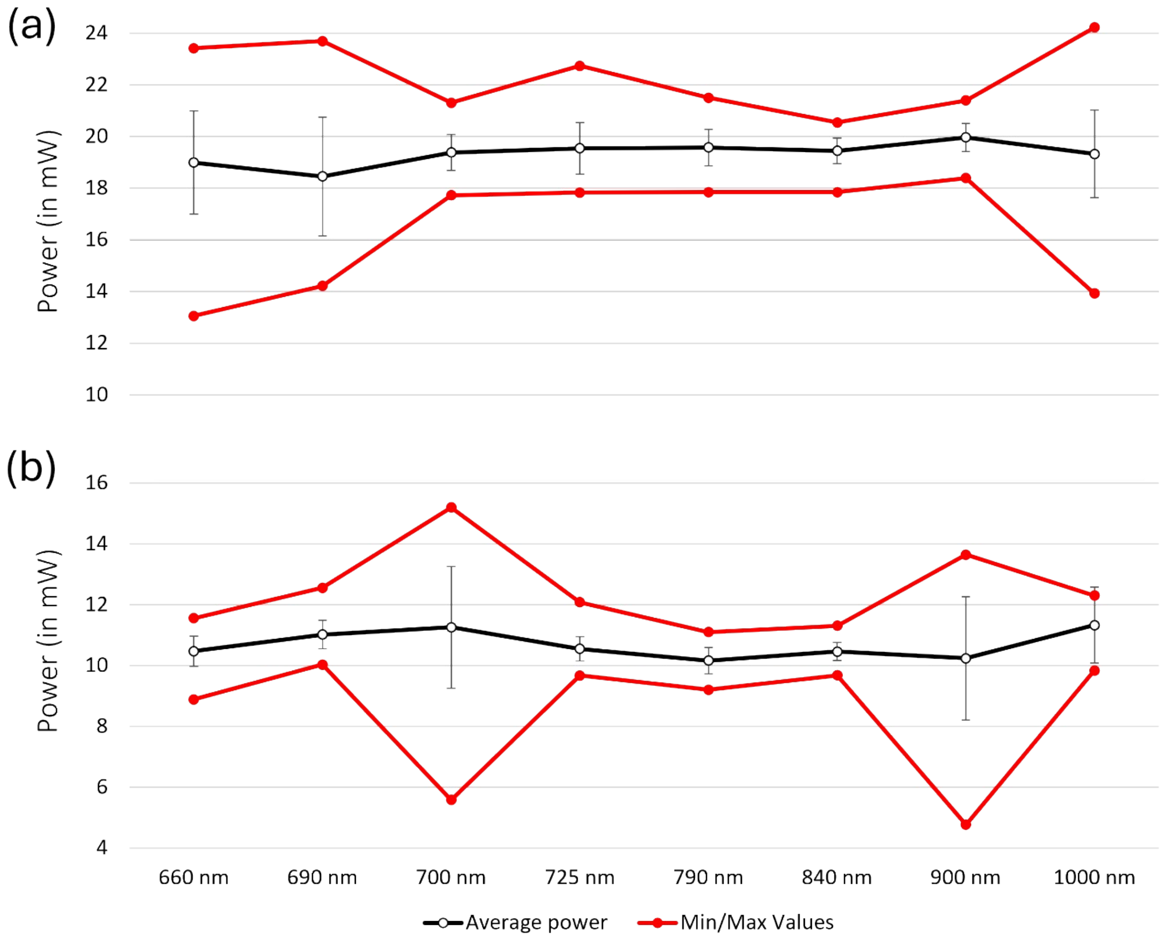

Figure S4. Power measurements as a function of the pulsed laser wavelengths used in (a) the 20 mW nanoheating experiment and (b) the 10 mW nanoheating experiment.

The Olympus objective has a high transmittance from 400 to 700 nm, which progressively falls above this range (see **Figure S5**). This difference was not accounted for during the experiments or the simulation work.

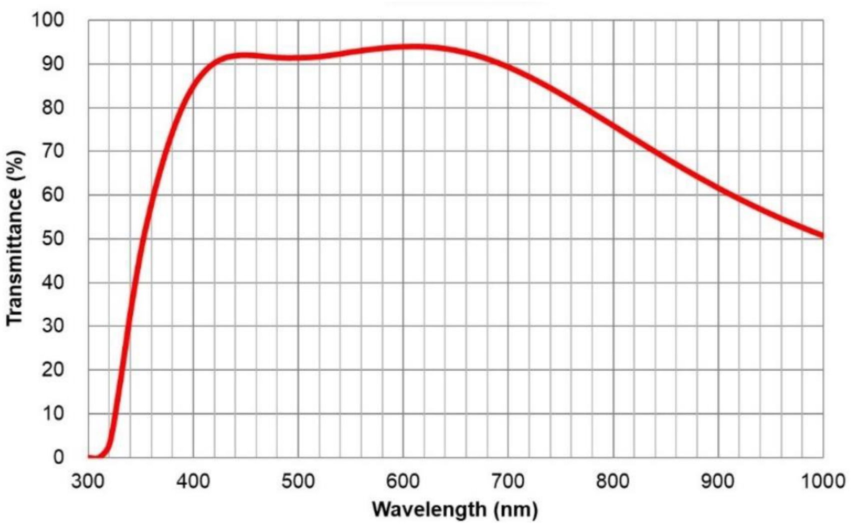

Figure S5. Transmittance of the Olympus UPlanFLN 10×/0.3 NA objective as a function of the wavelength of the incident light (from Olympus website).

Nanoheating Experiment 20 mW

SEM raw and colorized images

This section gathers tables containing six SEM images of five nanorods for all the different illumination wavelengths, for both the short and long rods. The raw SEM images, on the left, are compared to a version with colorized QDs in red dots to facilitate their identification and counting. The QDs considered in this study are only the ones situated on the top of the rods,

and of a size between 15 and 20 nm to avoid miscounting manufacturing defects of the rods as QDs. A summary table with the number of QDs per rod is presented at the end of this section, along with the average per wavelength and their respective standard deviation.

Short rods Unilluminated		
#	Raw	Colorized
1	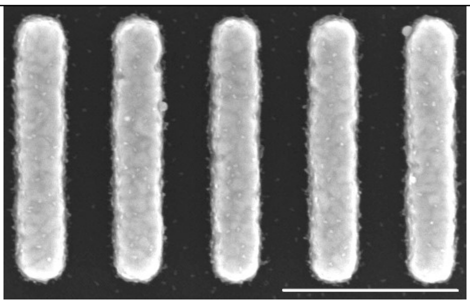	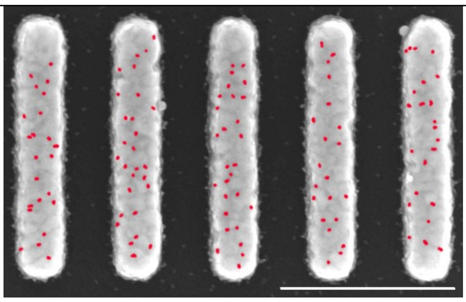
2	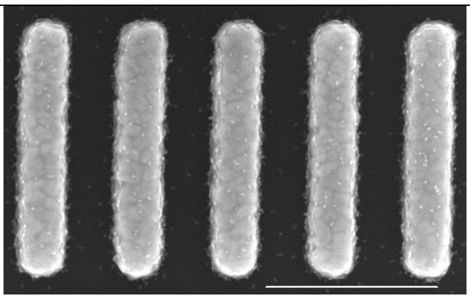	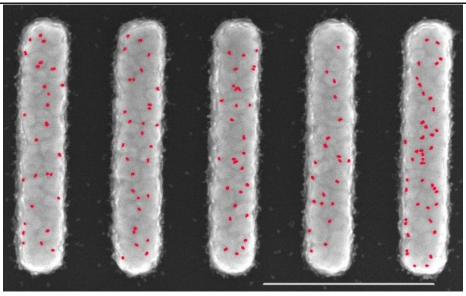

3	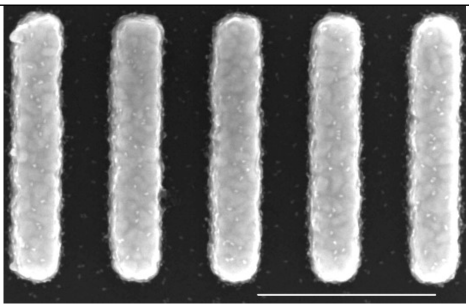	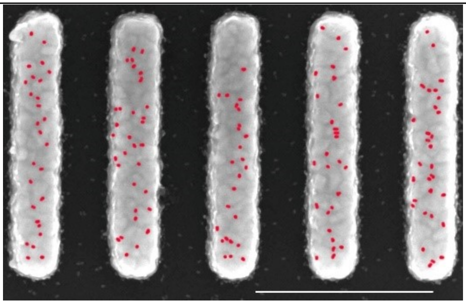
4	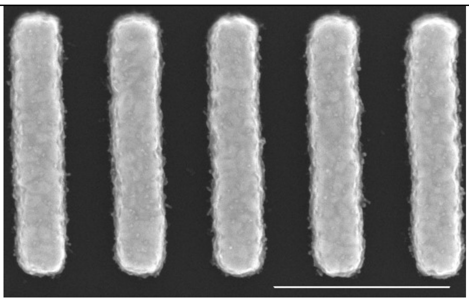	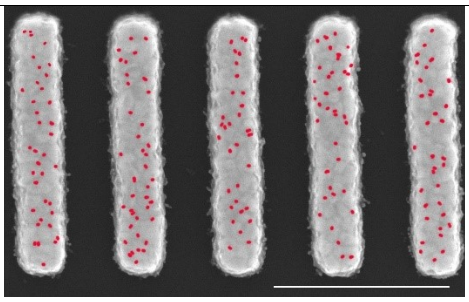
5	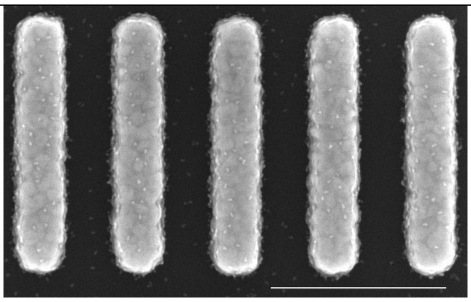	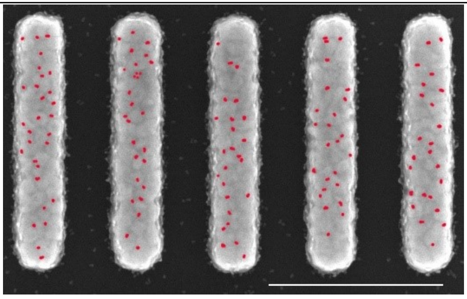
6	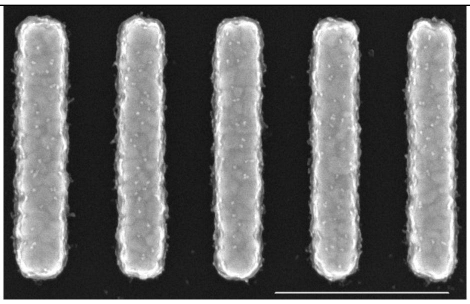	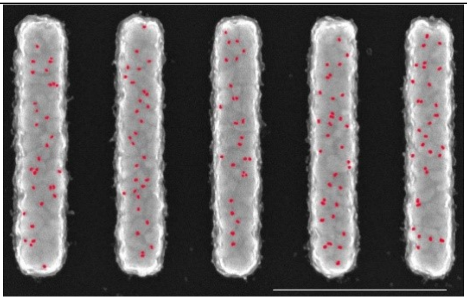
Short rods Illuminated at 660 nm / 0°		
#	Raw	Colorized
1	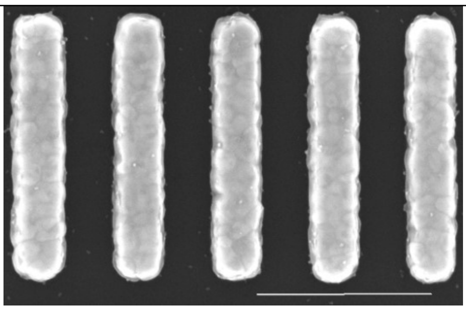	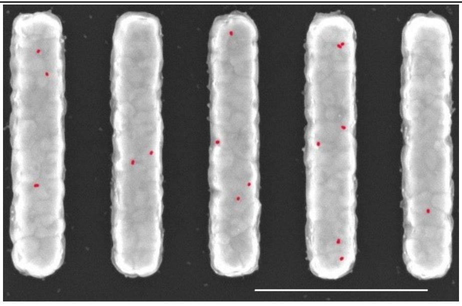

2	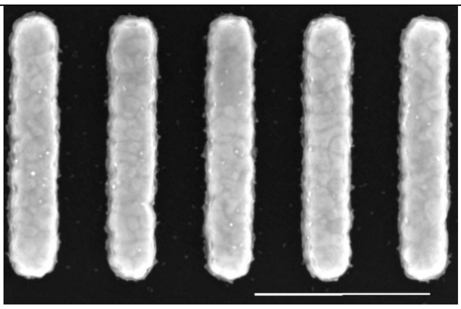	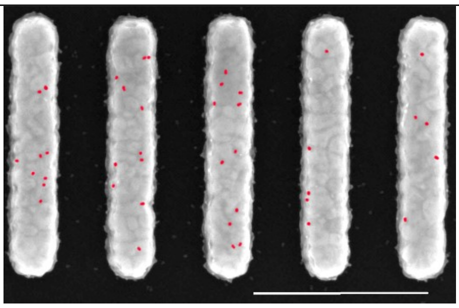
3	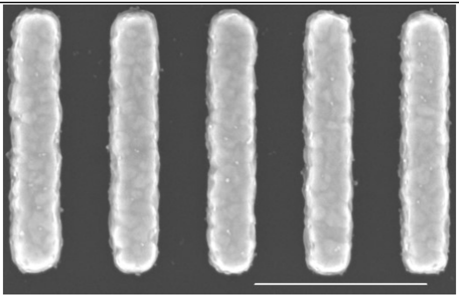	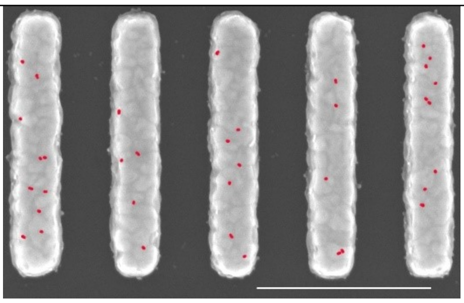
4	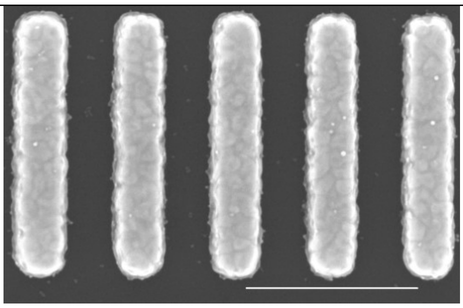	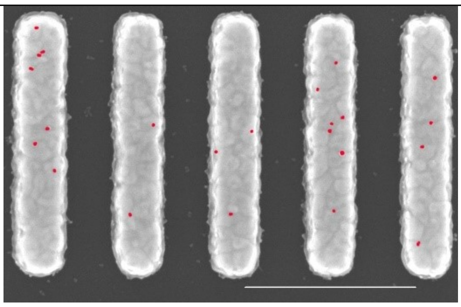
5	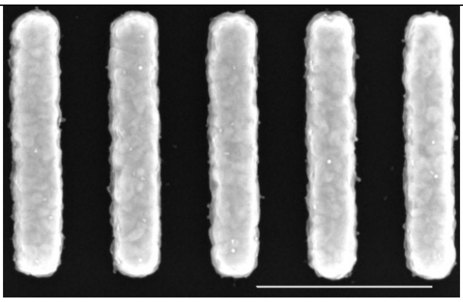	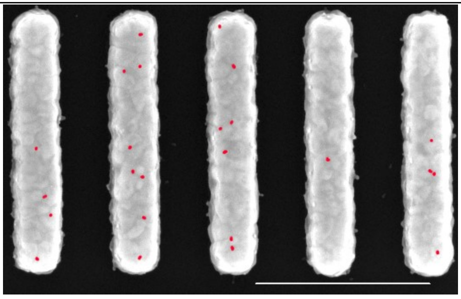
6	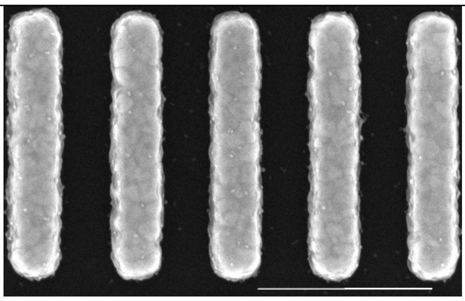	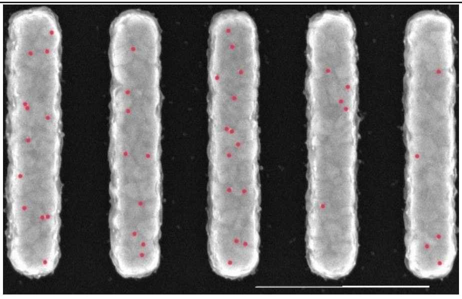
Short rods Illuminated at 690 nm / 0°		
#	Raw	Colorized

1	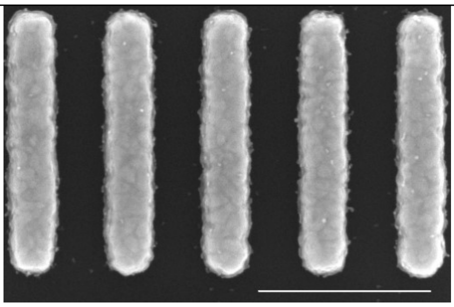	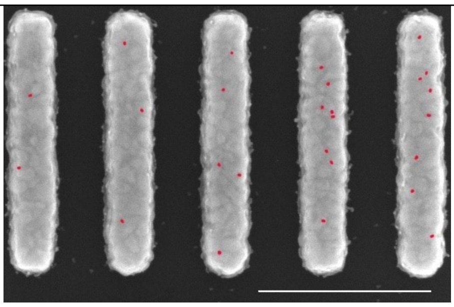
2	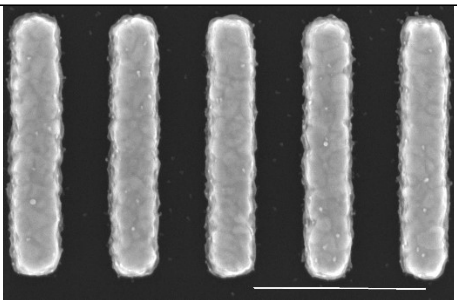	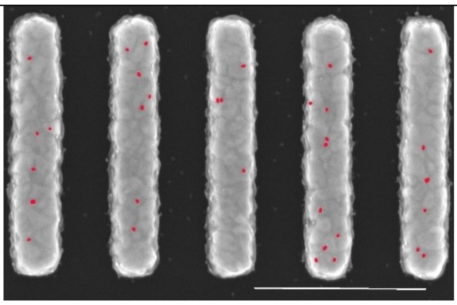
3	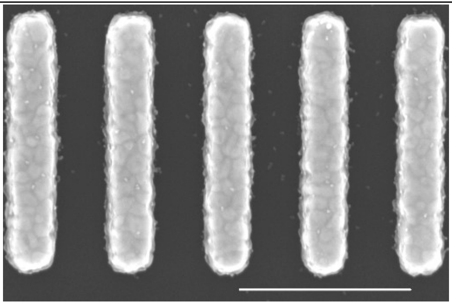	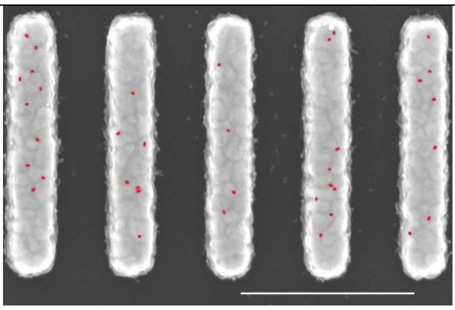
4	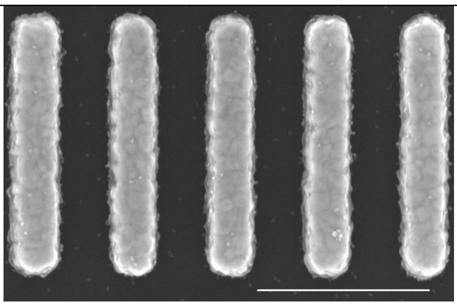	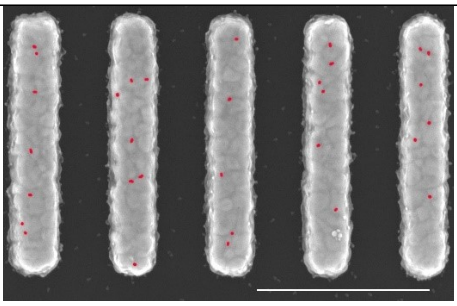
5	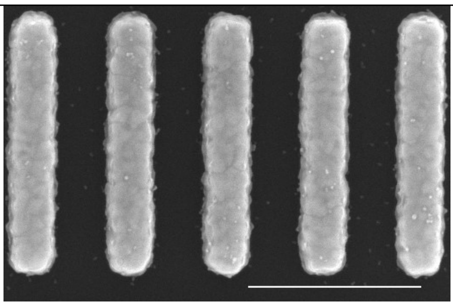	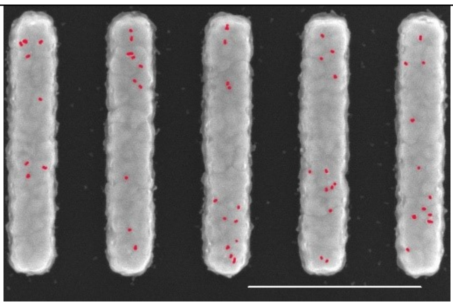

6	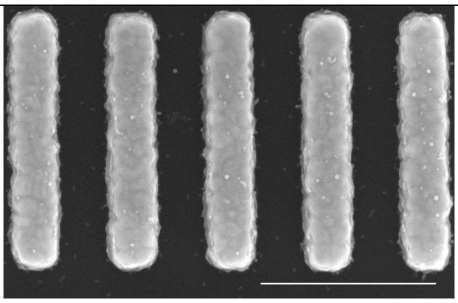	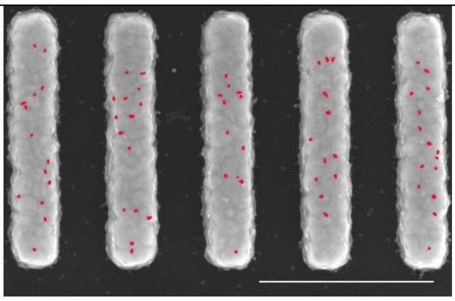
Short rods Illuminated at 725 nm / 0°		
#	Raw	Colorized
1	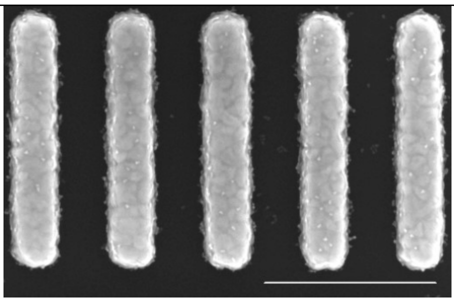	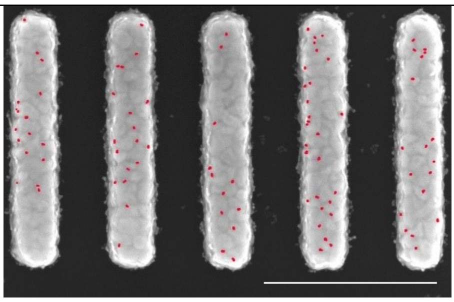
2	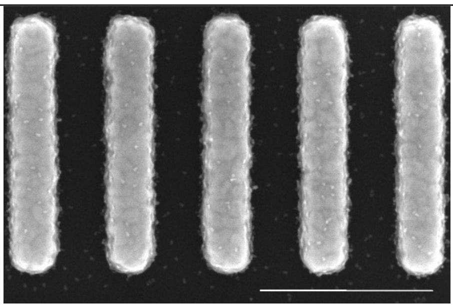	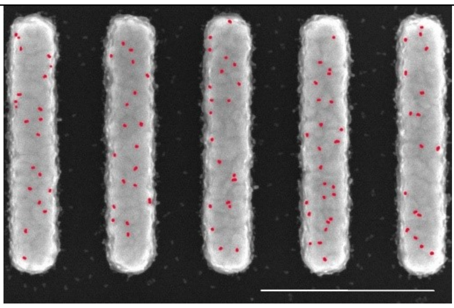
3	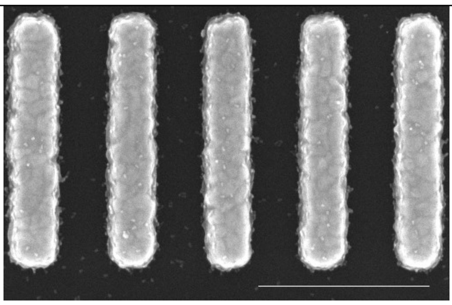	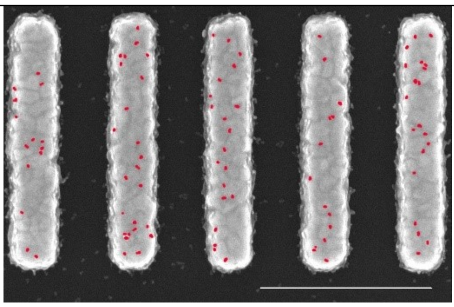
4	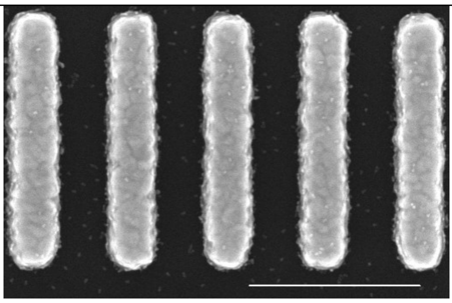	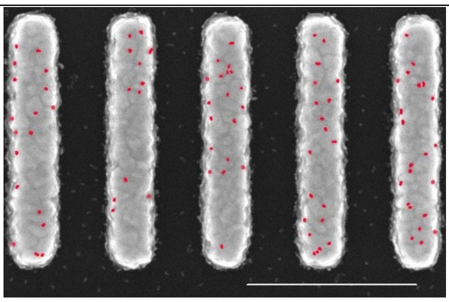

5	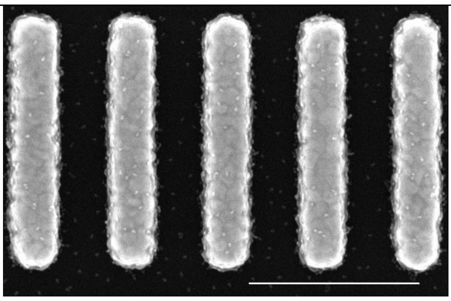	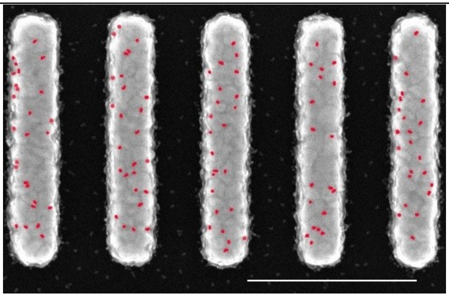
6	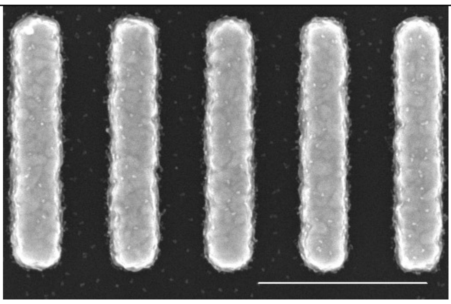	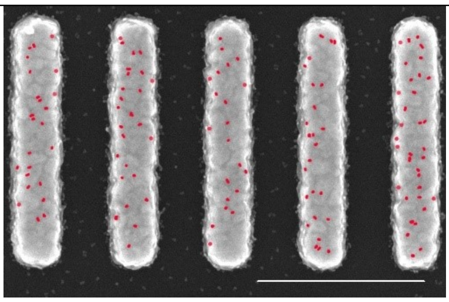
Short rods Illuminated at 840 / 0°		
#	Raw	Colorized
1	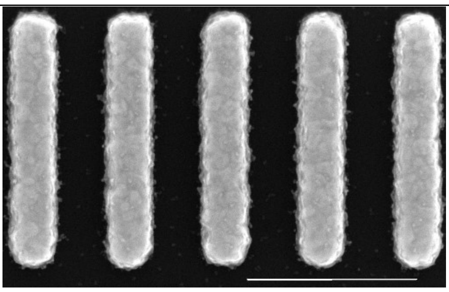	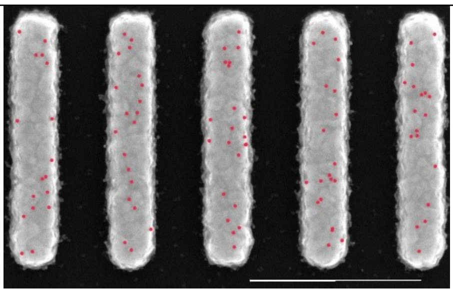
2	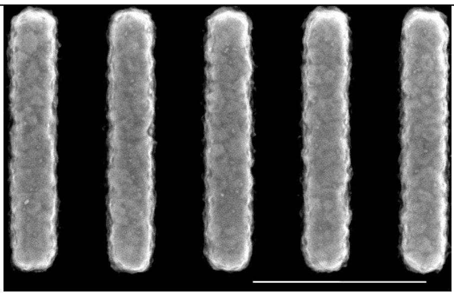	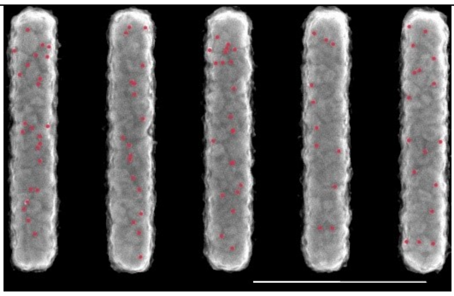
3	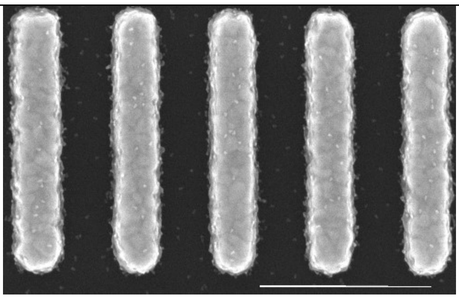	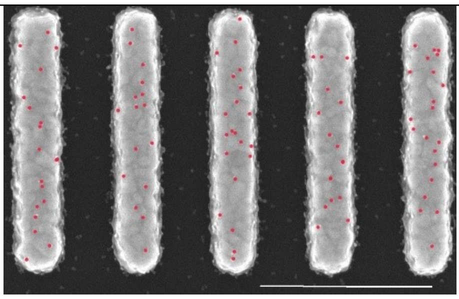

4	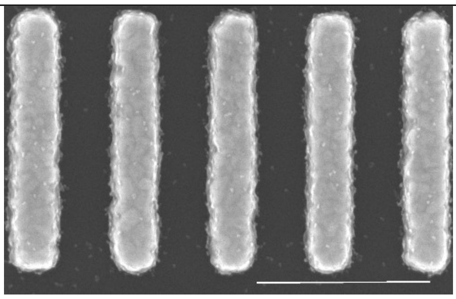	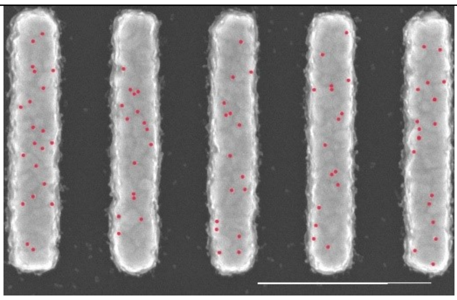
5	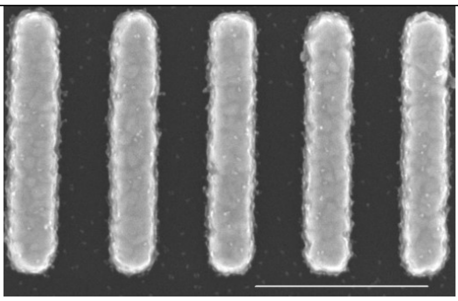	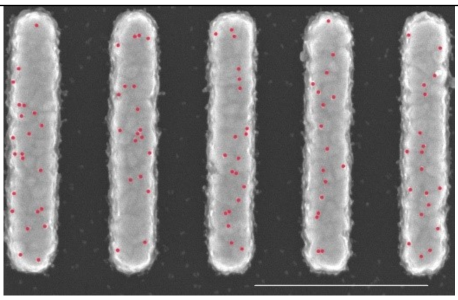
6	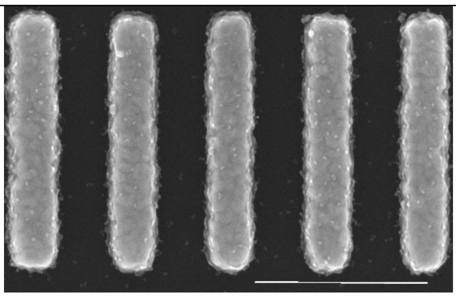	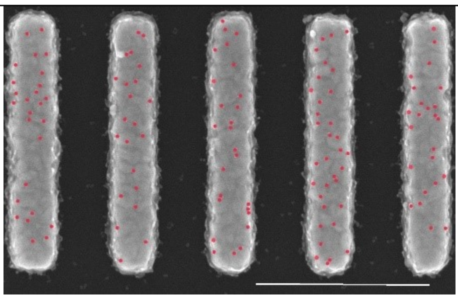

Short rods Illuminated at 900 nm / 0°		
#	Raw	Colorized
1	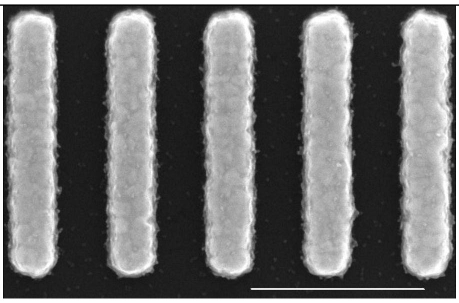	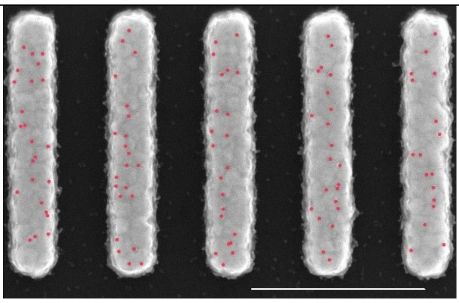
2	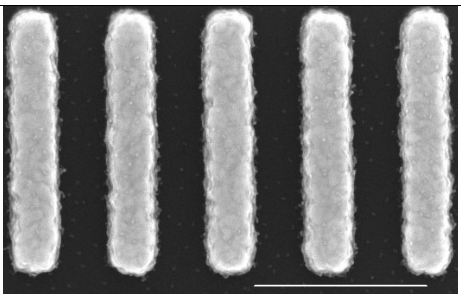	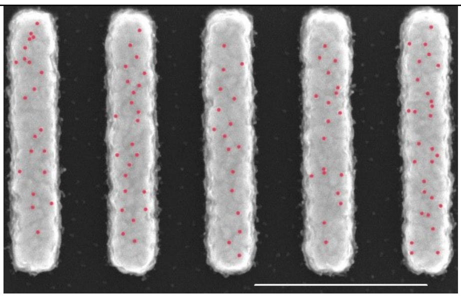
3	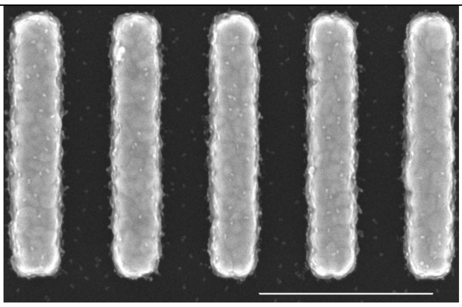	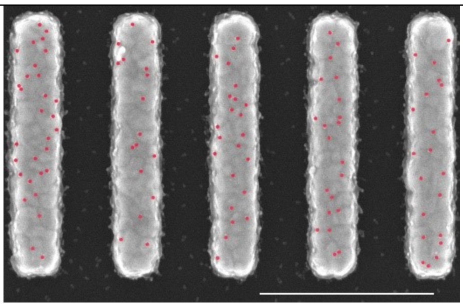
4	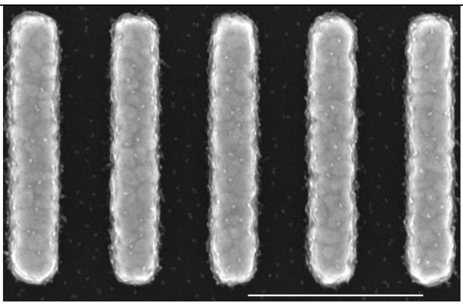	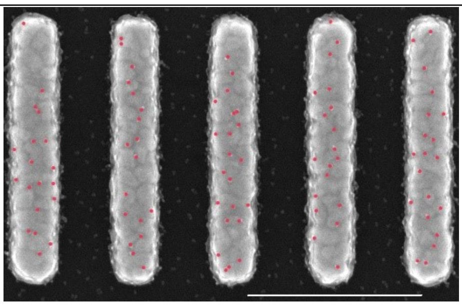
5	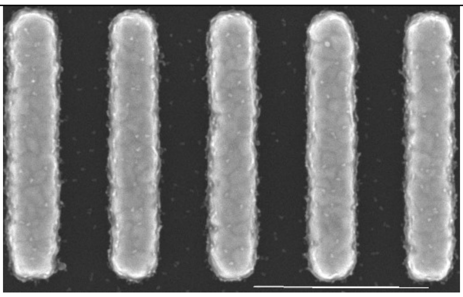	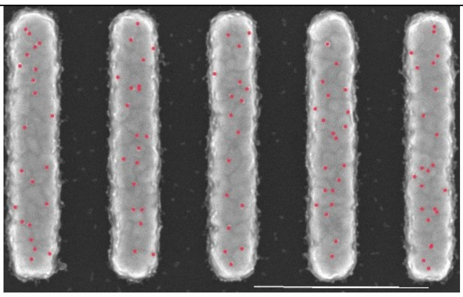

6	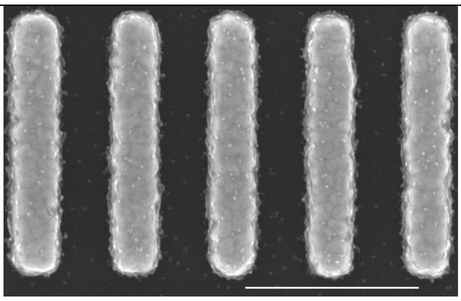	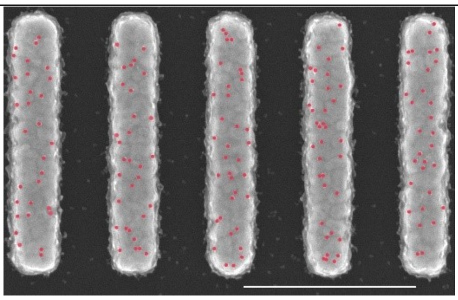
Short rods Illuminated 660 nm / 90°		
#	Raw	Colorized
1	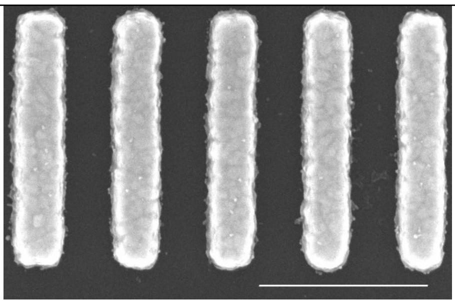	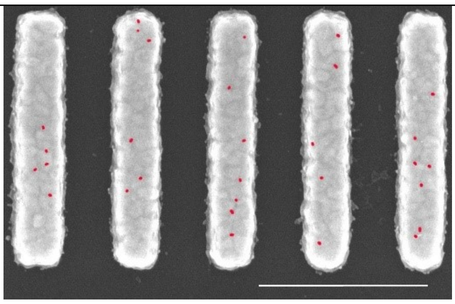
2	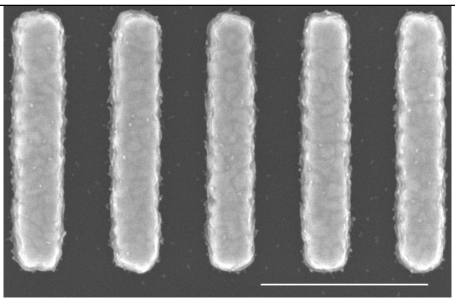	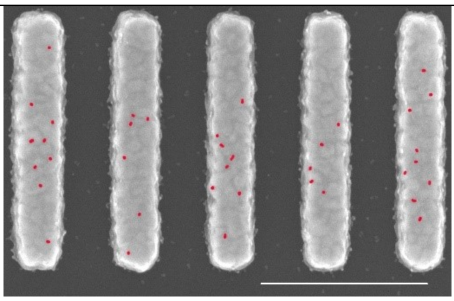
3	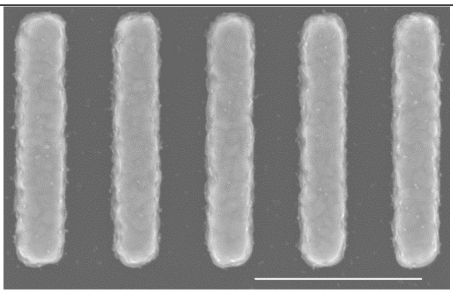	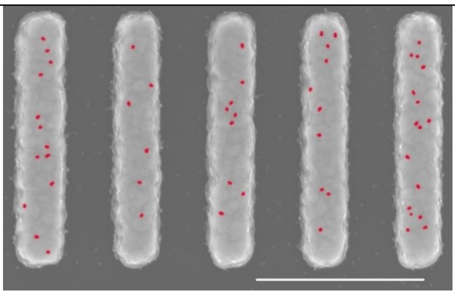
4	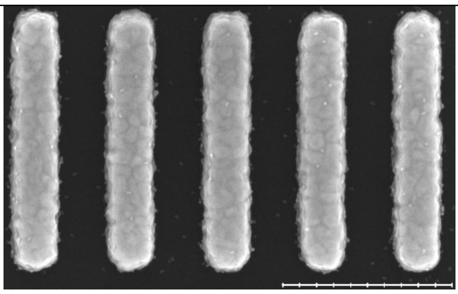	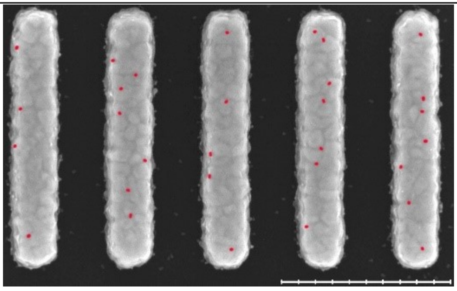

5	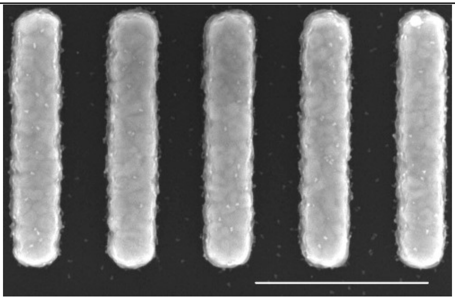	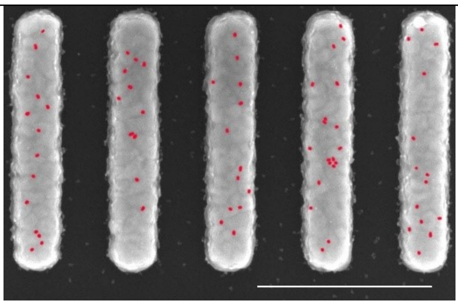
6	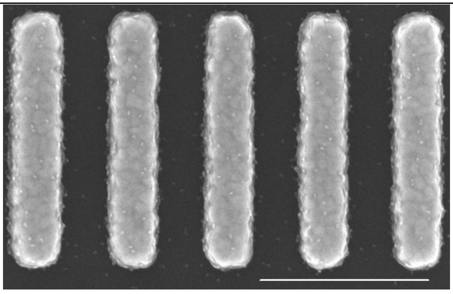	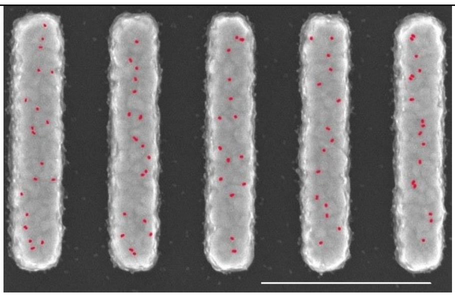

Short rods Illuminated at 700 nm / 90°		
#	Raw	Colorized
1	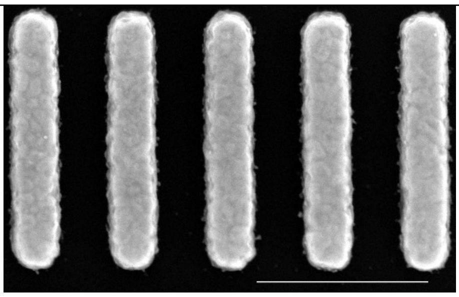	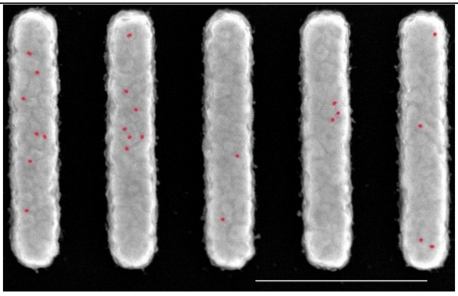
2	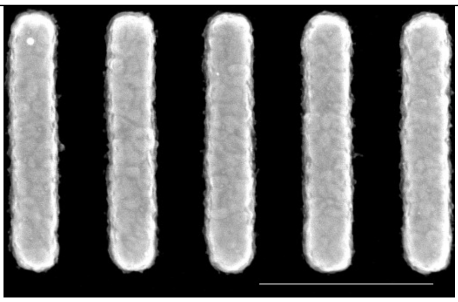	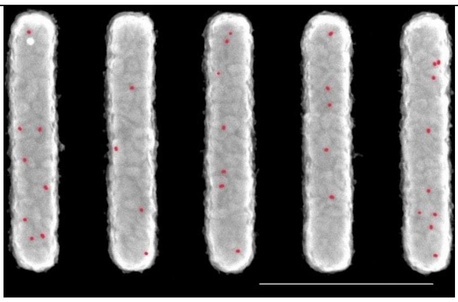

3	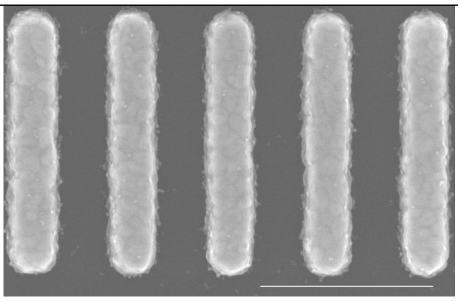	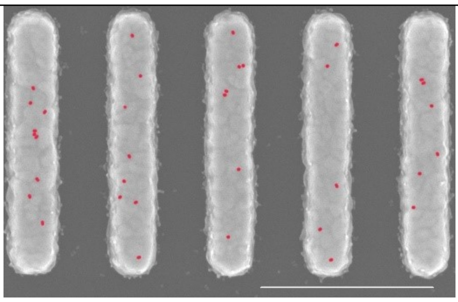
4	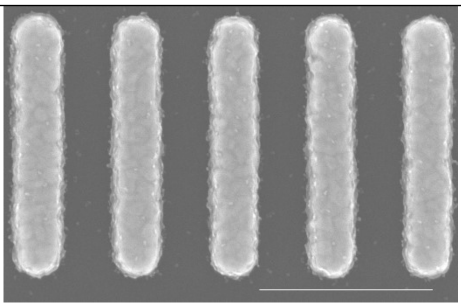	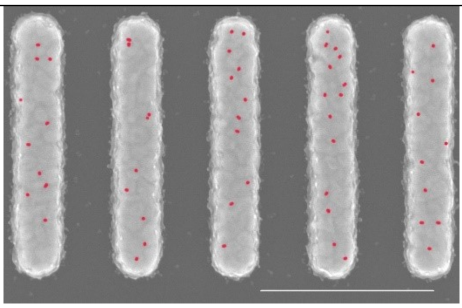
5	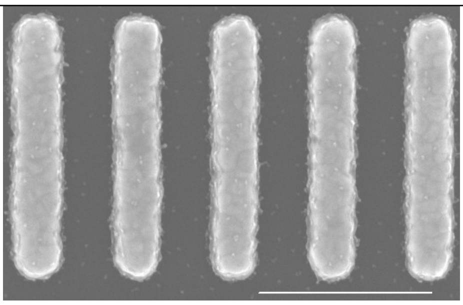	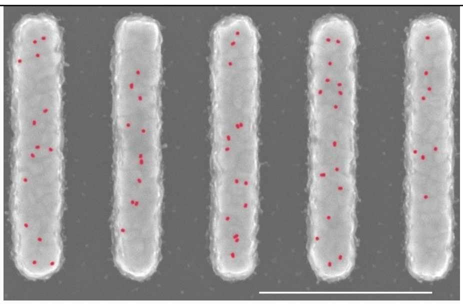
6	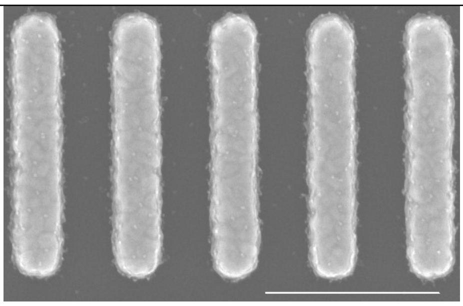	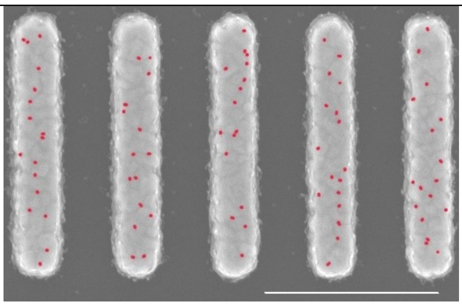
Short rods Illuminated at 840 nm / 90°		
#	Raw	Colorized
1	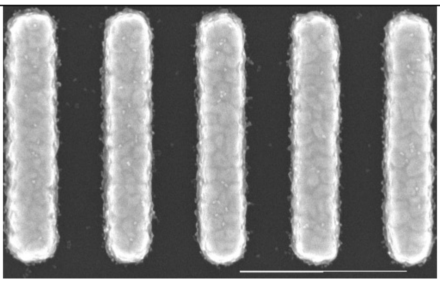	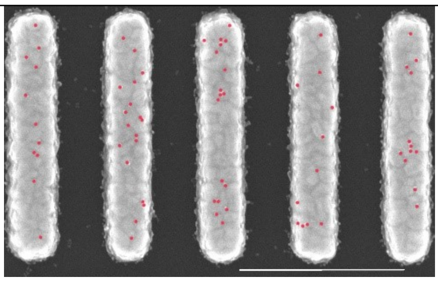

2	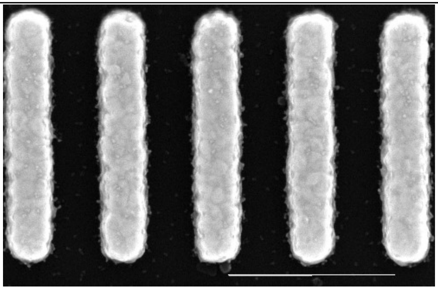	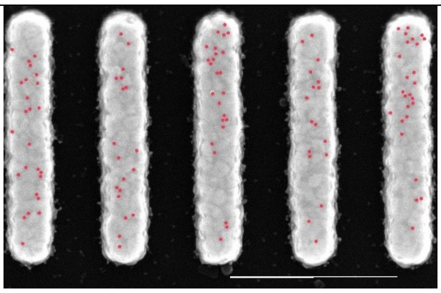
3	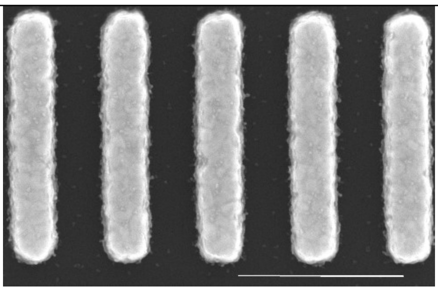	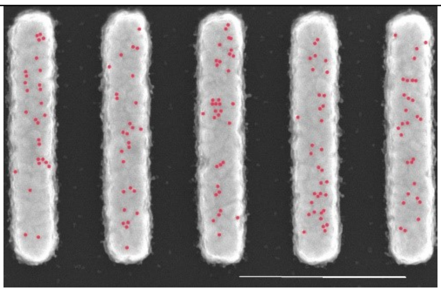
4	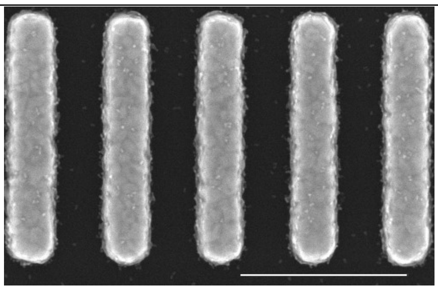	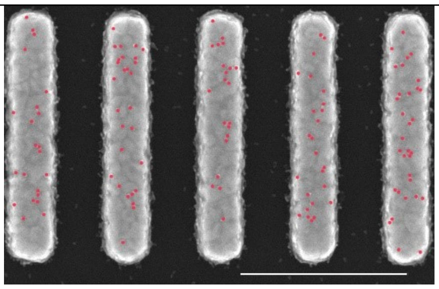
5	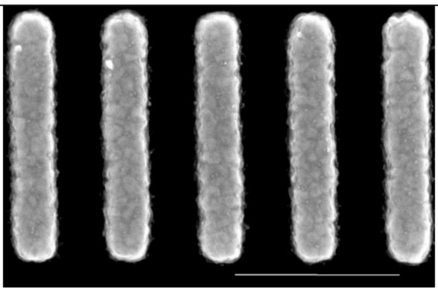	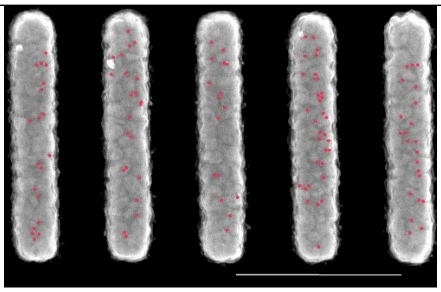
6	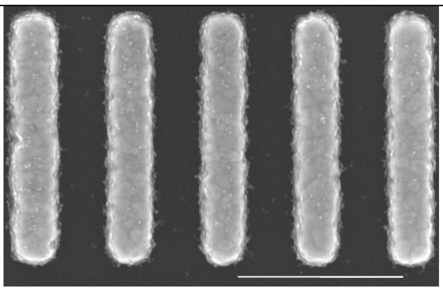	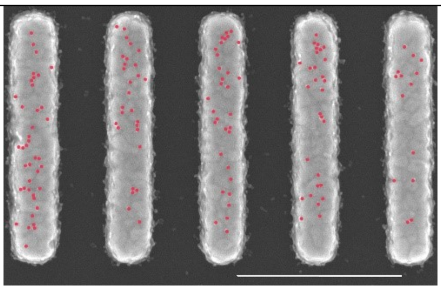

Short rods Illuminated at 1000 nm / 90°		
#	Raw	Colorized

1	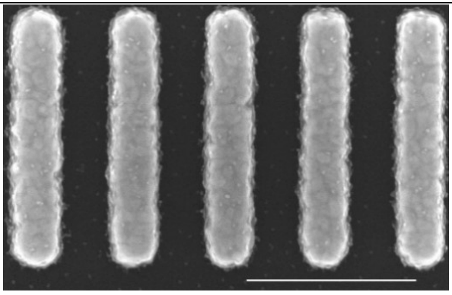	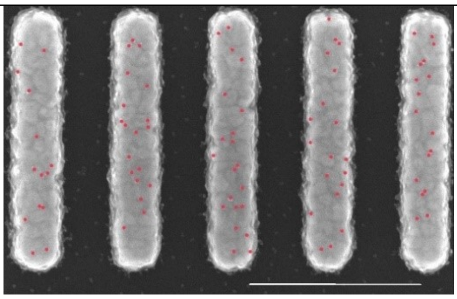
2	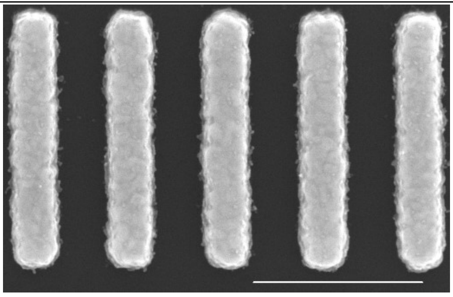	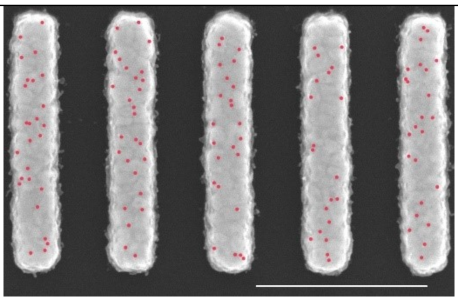
3	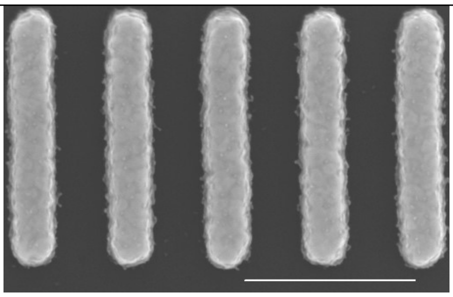	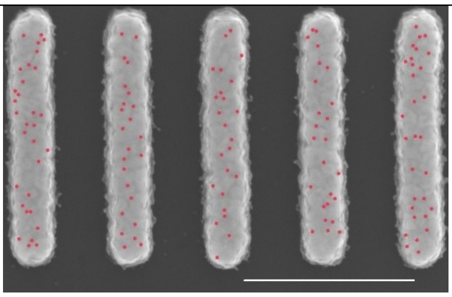
4	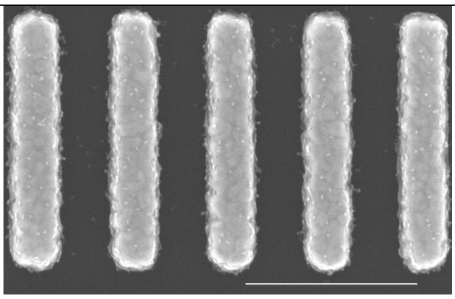	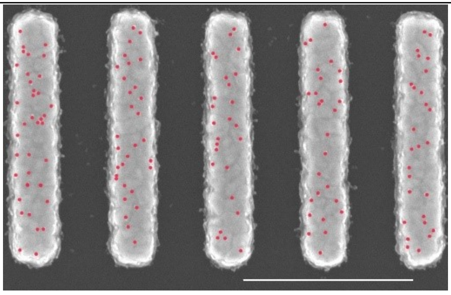
5	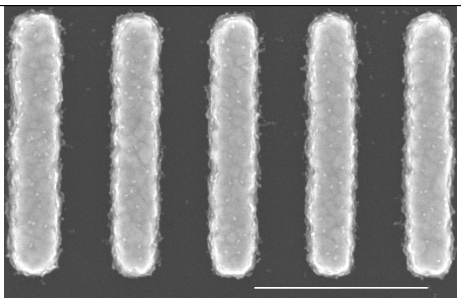	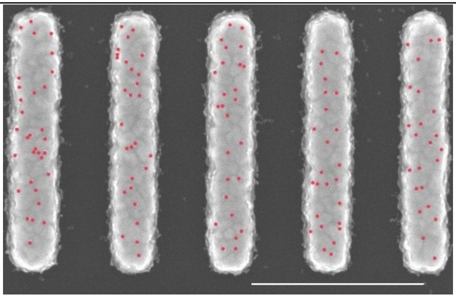

6	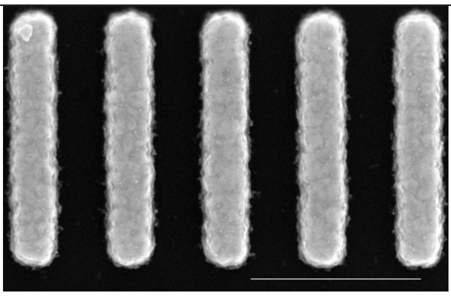	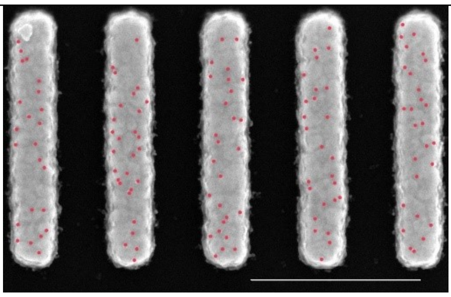

Long rods Unilluminated		
#	Raw	Colorized
1	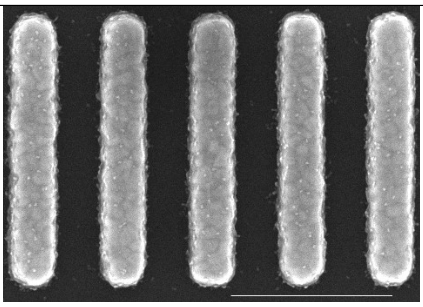	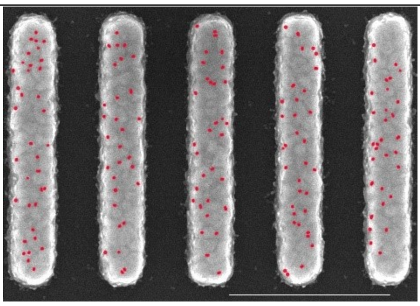
2	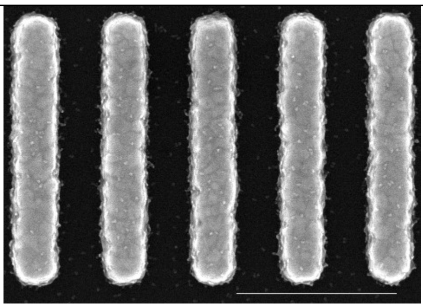	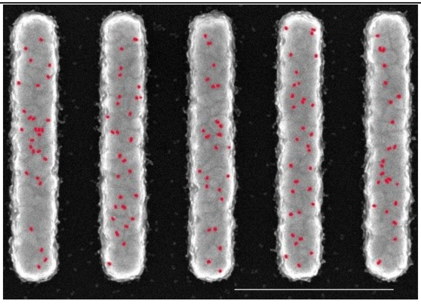
3	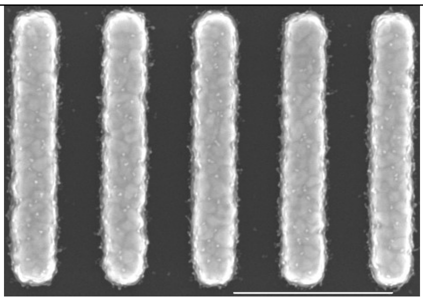	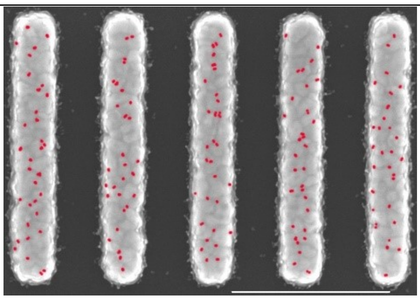

4	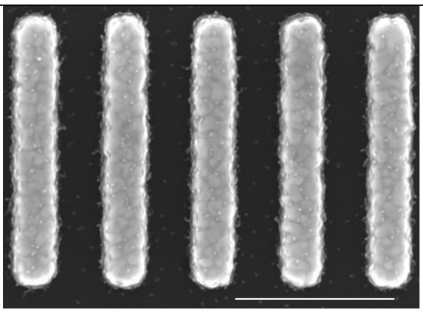	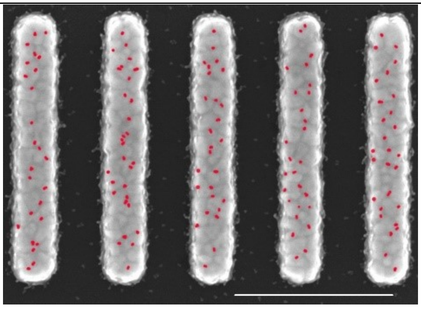
5	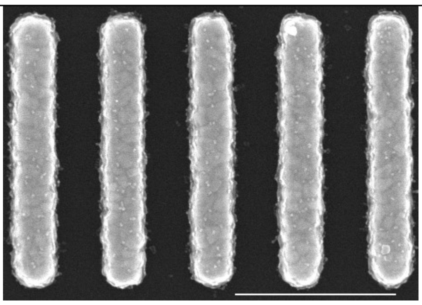	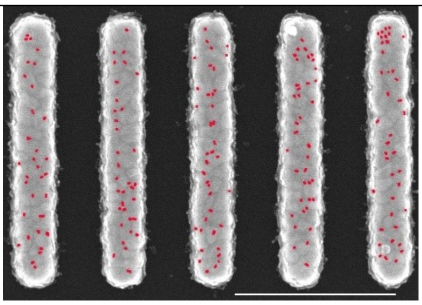
6	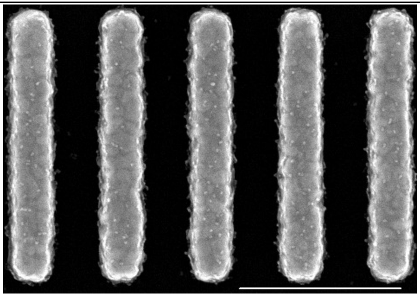	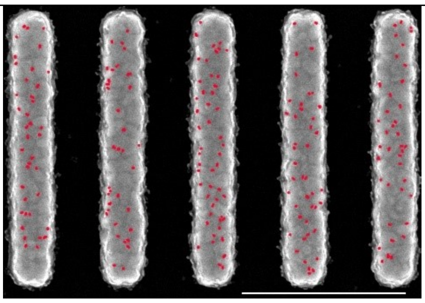
Long rods Illuminated at 660 nm / 0°		
#	Raw	Colorized
1	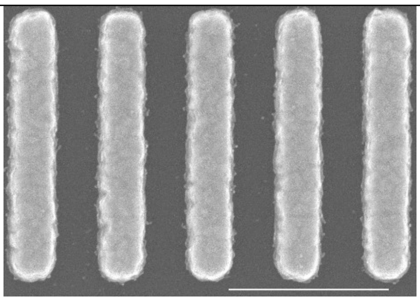	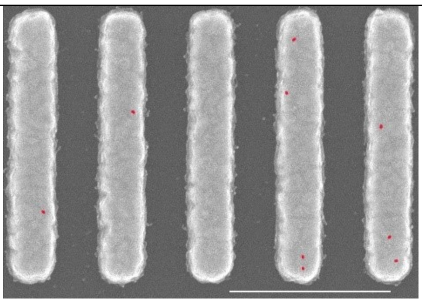
2	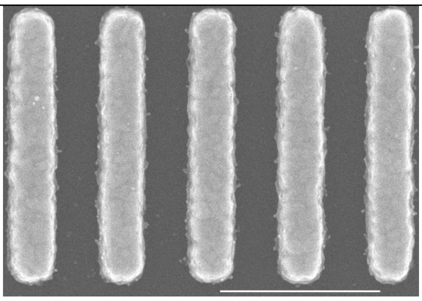	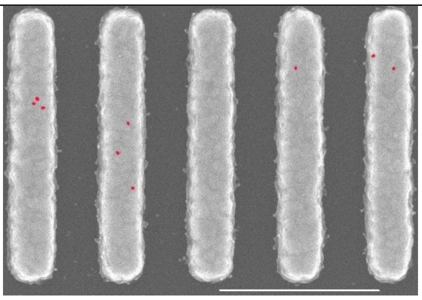

<table>
<tr><td>3</td><td>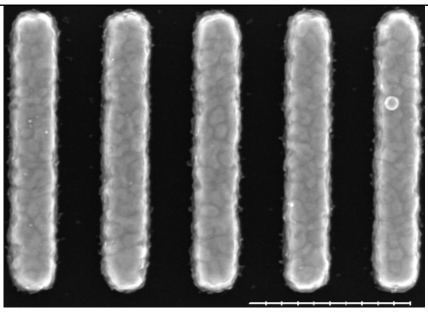</td><td>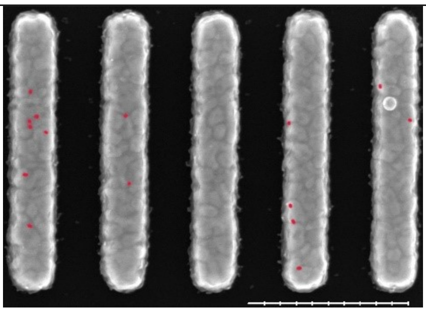</td></tr>
<tr><td>4</td><td>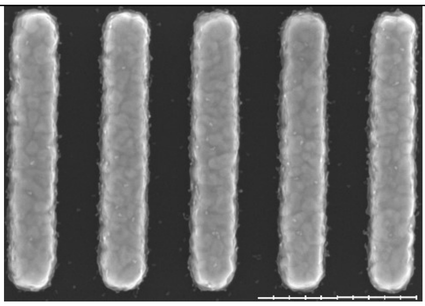</td><td>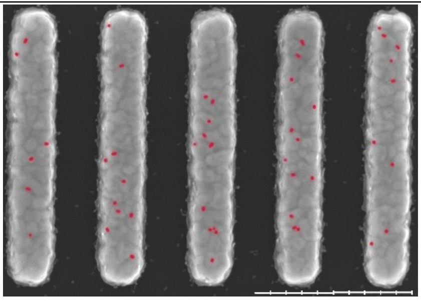</td></tr>
<tr><td>5</td><td>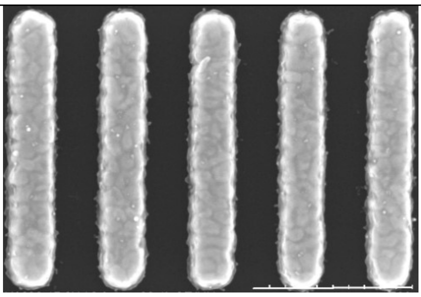</td><td>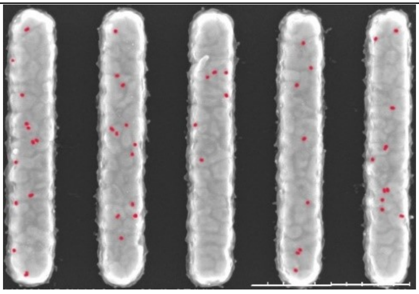</td></tr>
<tr><td>6</td><td>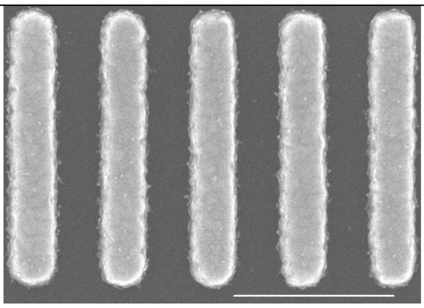</td><td>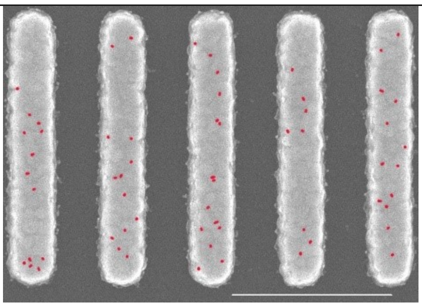</td></tr>
<tr><td colspan="3">Long rods Illuminated at 725 nm / 0°</td></tr>
<tr><td>#</td><td>Raw</td><td>Colorized</td></tr>
<tr><td>1</td><td>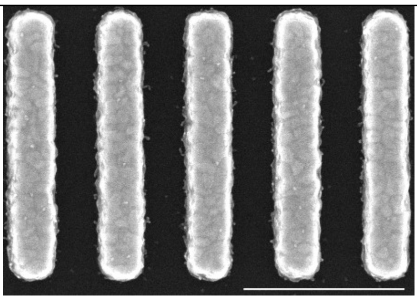</td><td>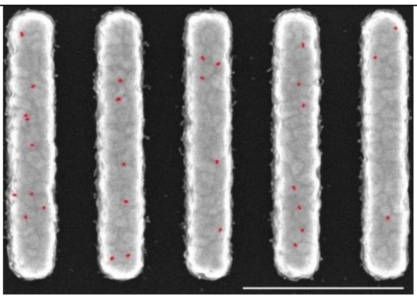</td></tr>
</table>

2	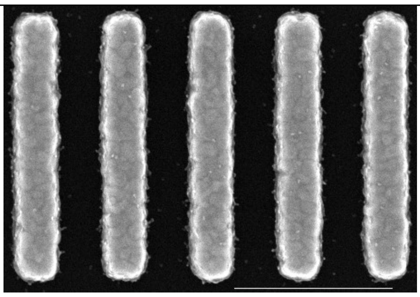	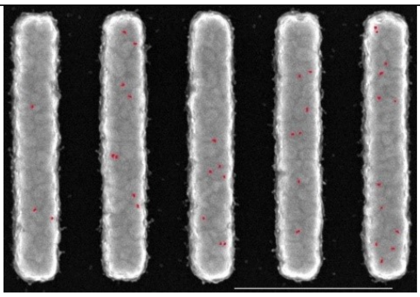
3	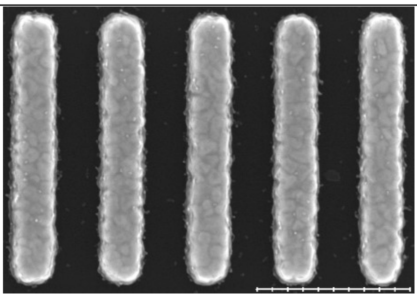	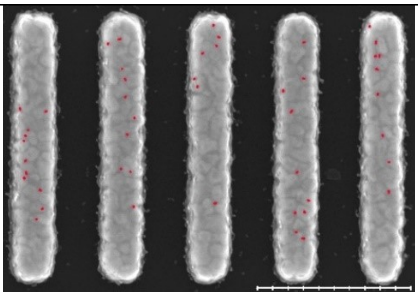
4	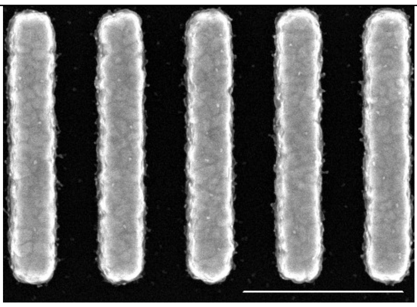	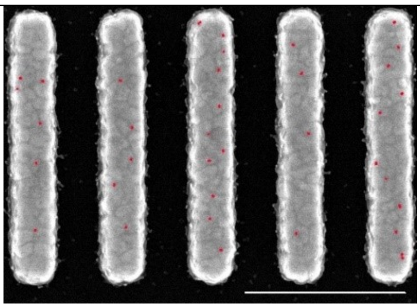
5	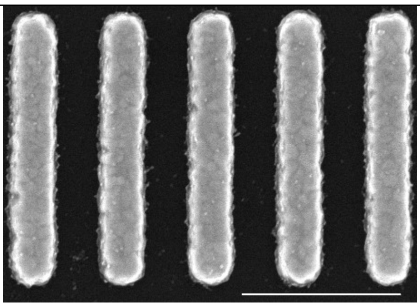	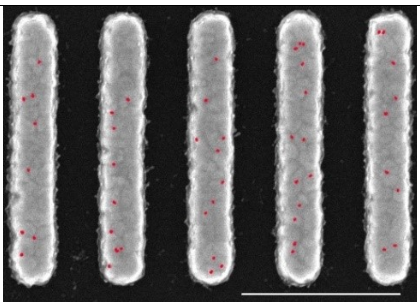
6	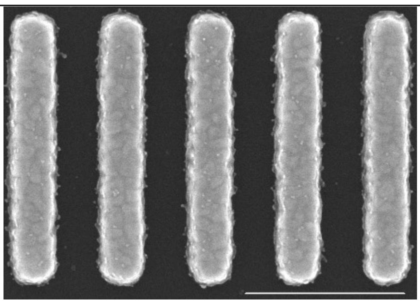	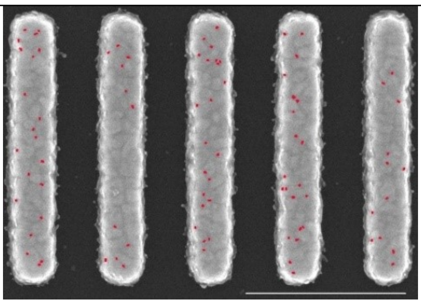
Long rods Illuminated at 790 nm / 0°		
#	Raw	Colorized

1	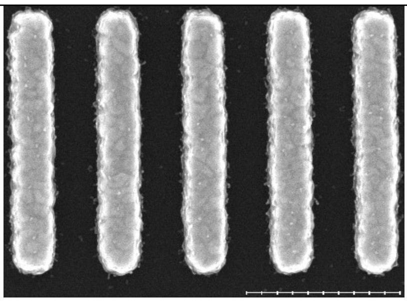	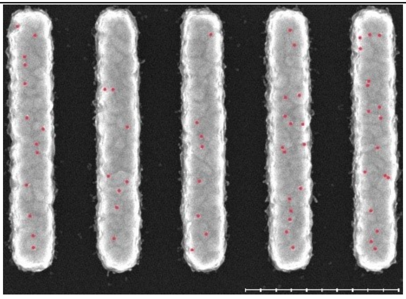
2	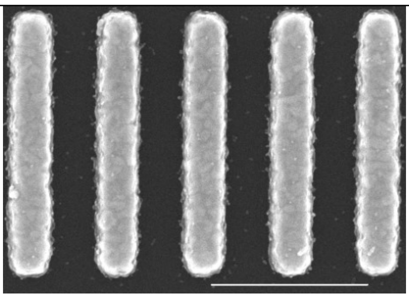	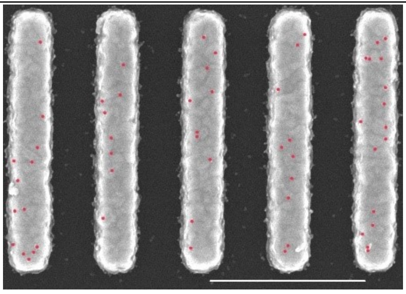
3	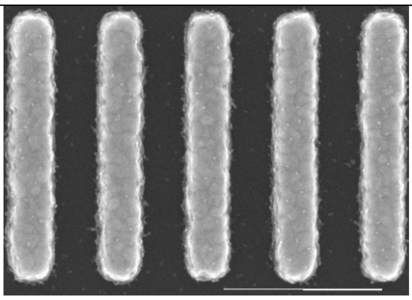	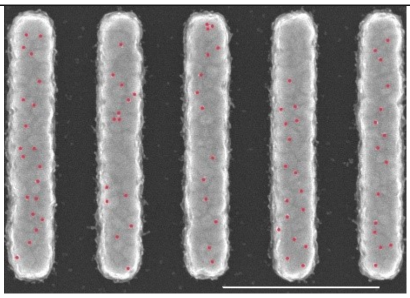
4	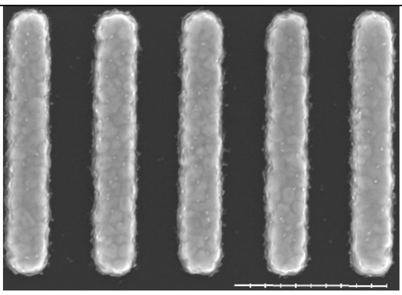	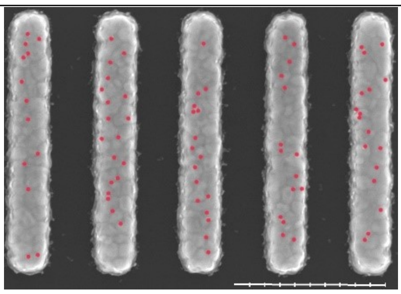
5	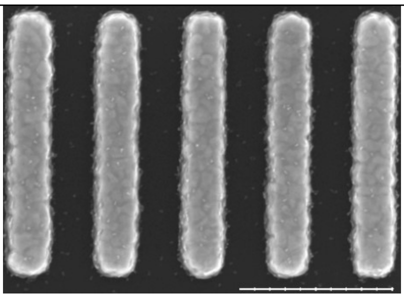	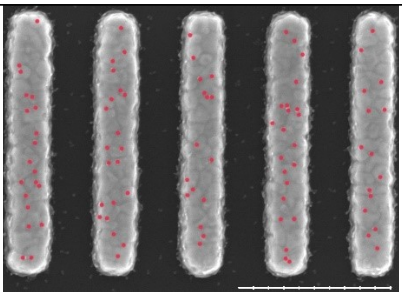

6	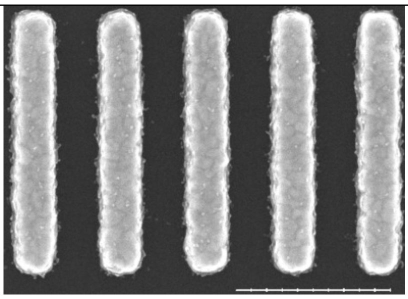	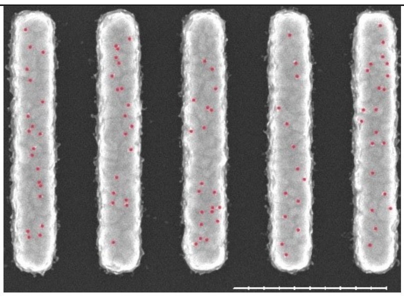

Long rods Illuminated at 840 nm / 0°		
#	Raw	Colorized
1	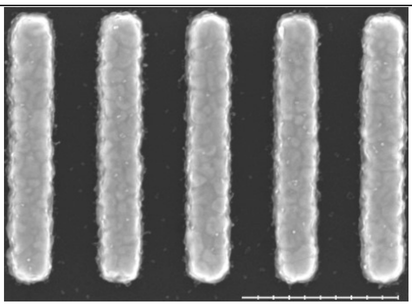	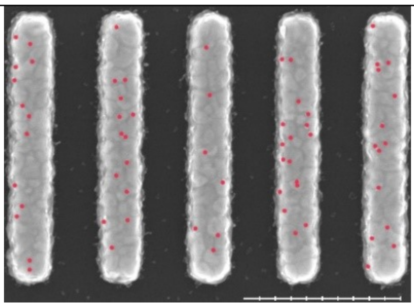
2	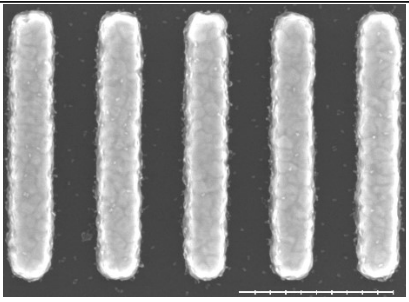	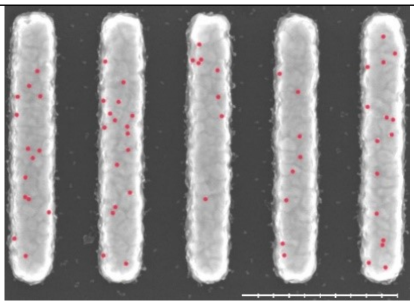
3	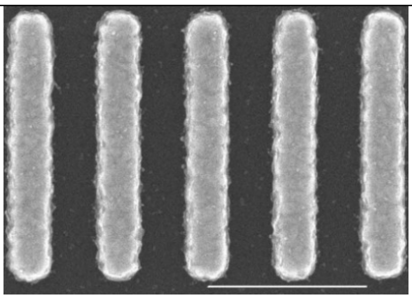	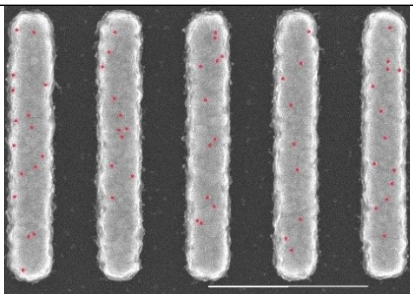

4	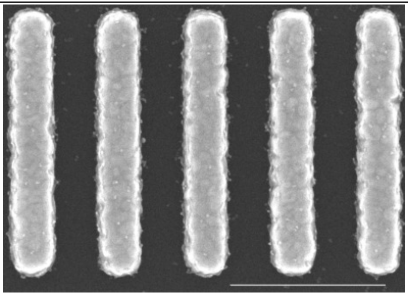	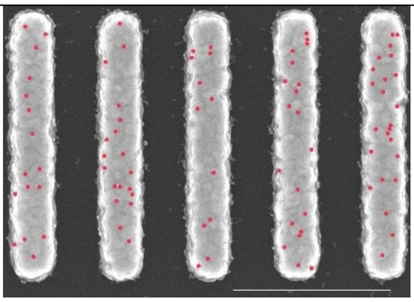
5	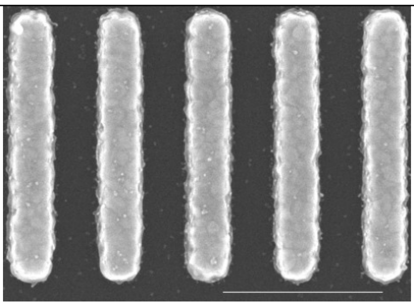	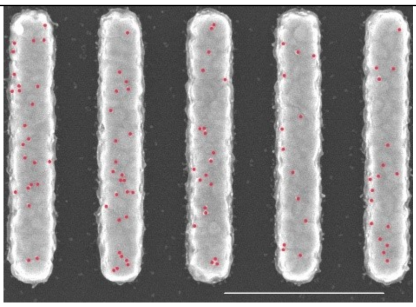
6	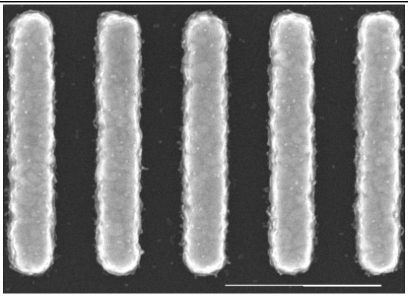	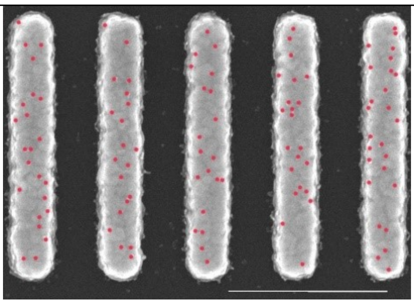
Long rods Illuminated at 900 nm / 0°		
#	Raw	Colorized
1	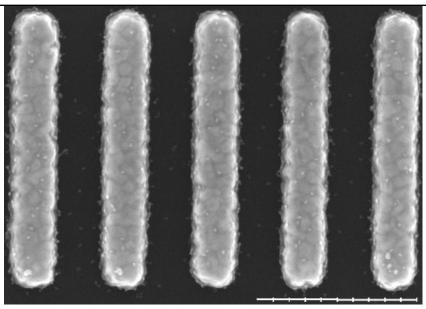	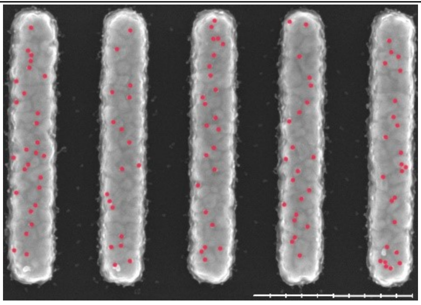
2	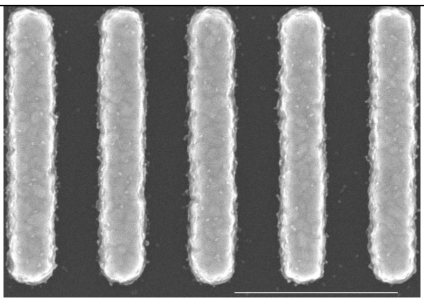	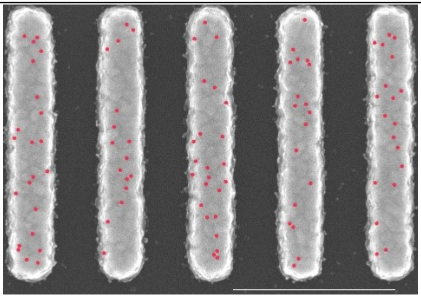

<table>
<tr><td>3</td><td>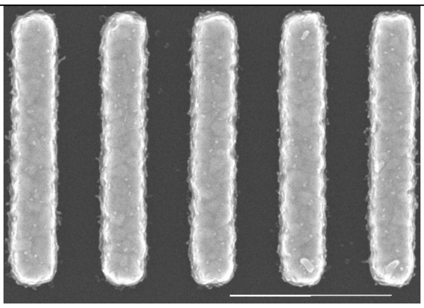</td><td>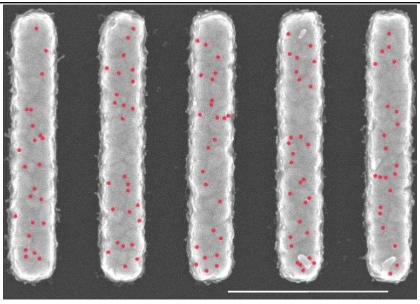</td></tr>
<tr><td>4</td><td>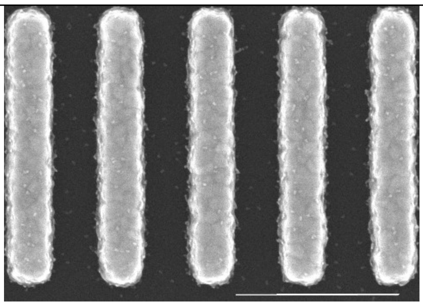</td><td>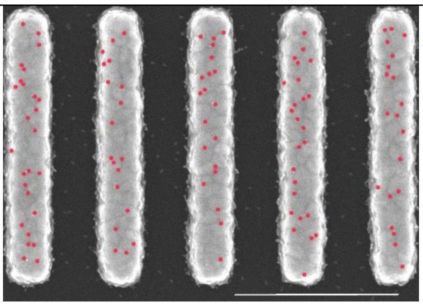</td></tr>
<tr><td>5</td><td>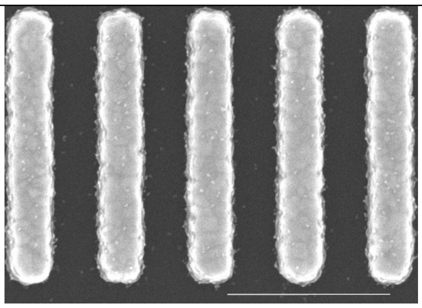</td><td>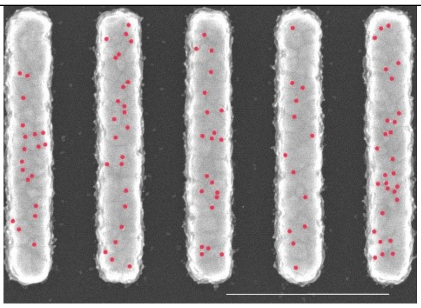</td></tr>
<tr><td>6</td><td>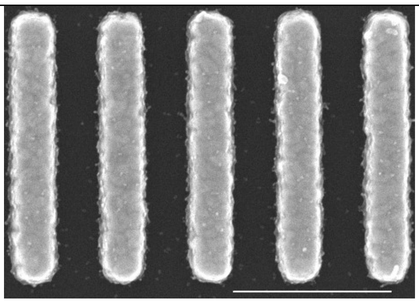</td><td>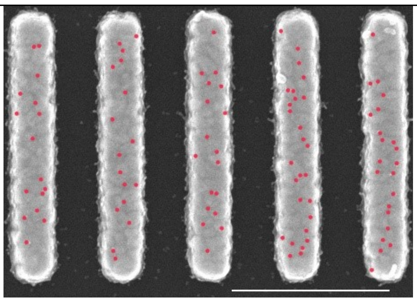</td></tr>
<tr><td colspan="3">Long rods Illuminated at 660 nm / 90°</td></tr>
<tr><td>#</td><td>Raw</td><td>Colorized</td></tr>
<tr><td>1</td><td>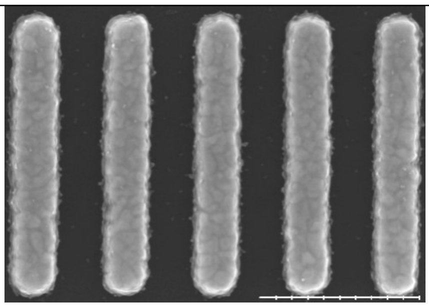</td><td>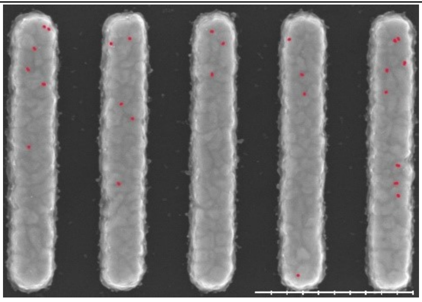</td></tr>
</table>

2	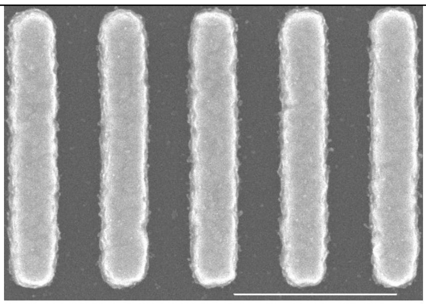	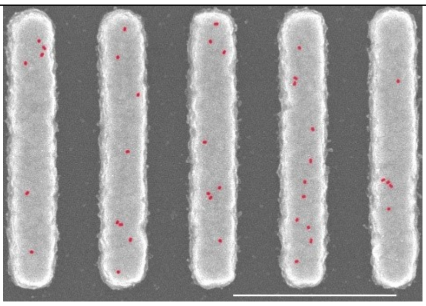
3	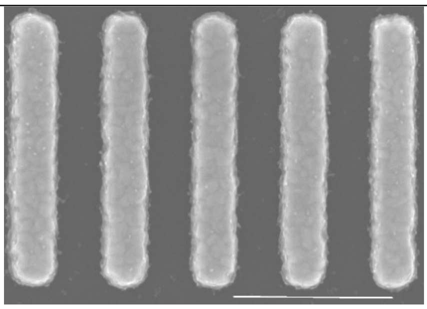	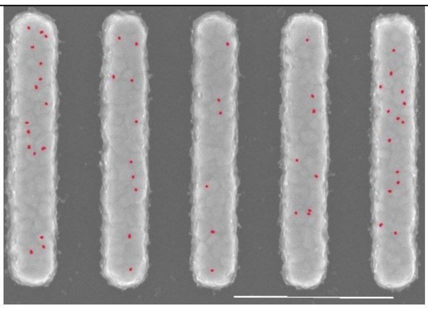
4	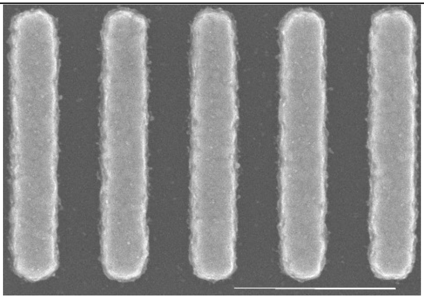	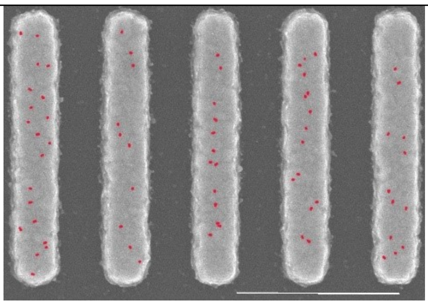
5	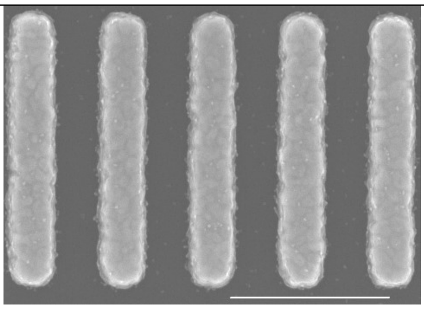	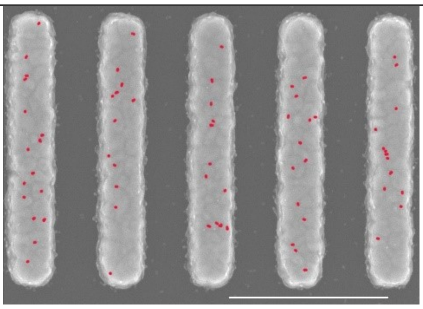
6	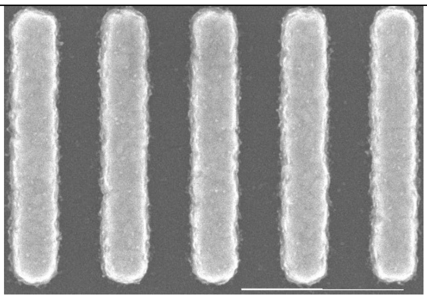	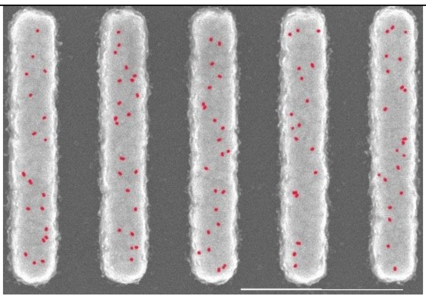
Long rods Illuminated at 700 nm / 90°		
#	Raw	Colorized

1	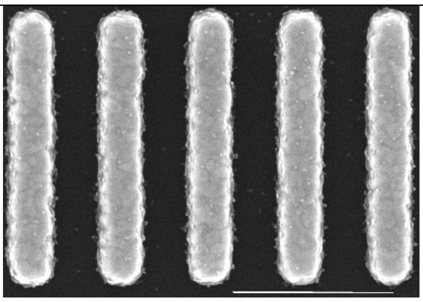	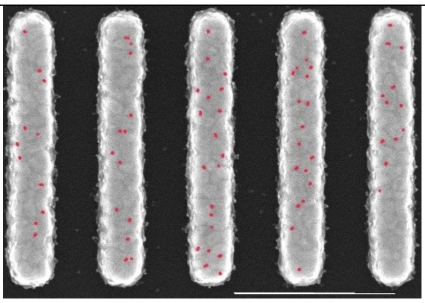
2	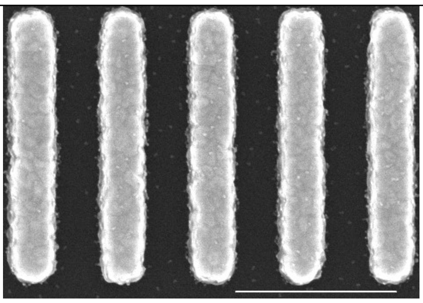	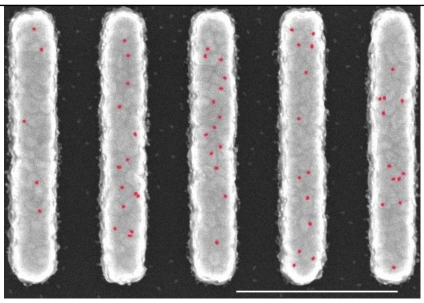
3	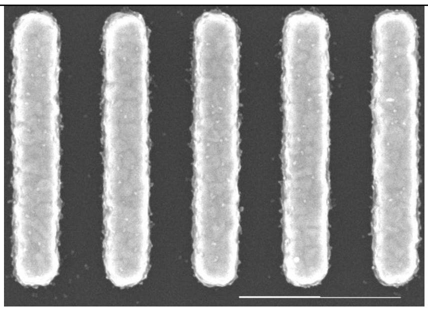	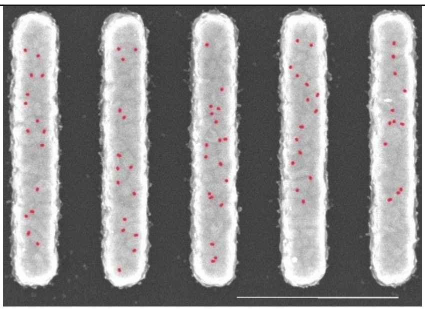
4	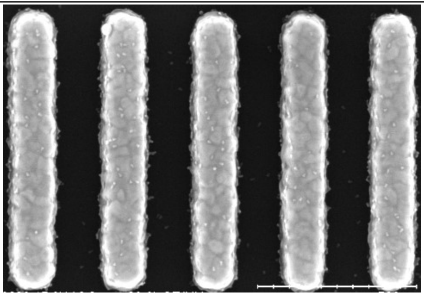	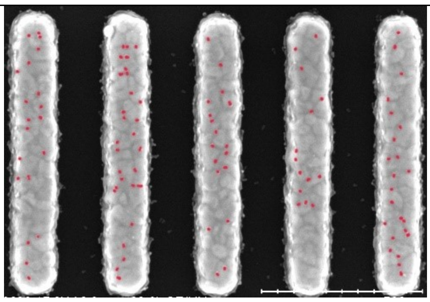
5	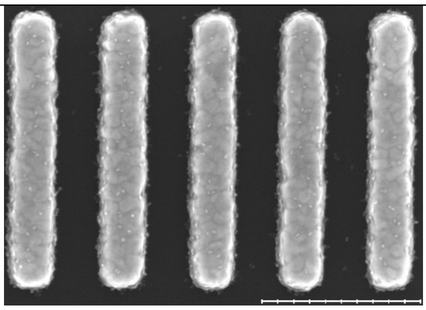	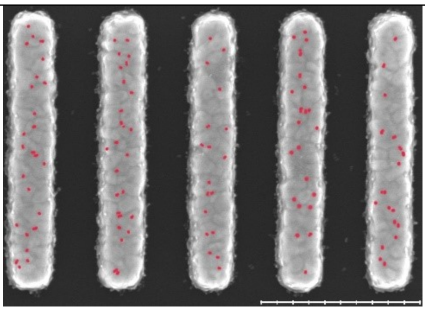

6	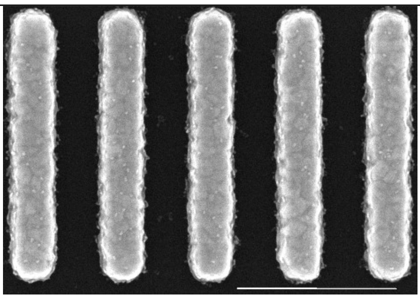	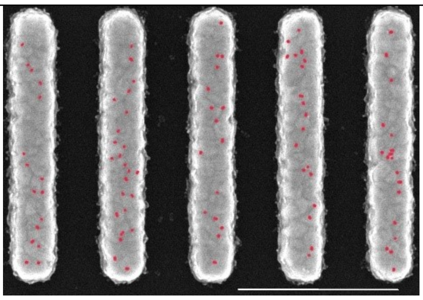
Long rods Illuminated at 840 nm / 90°		
#	Raw	Colorized
1	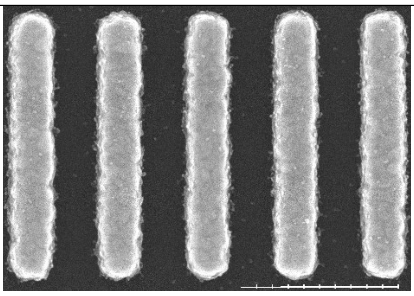	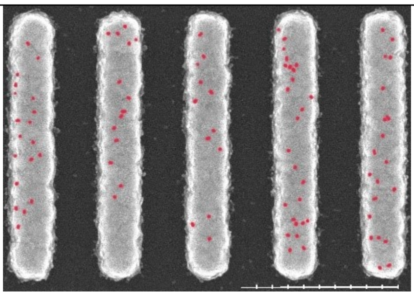
2	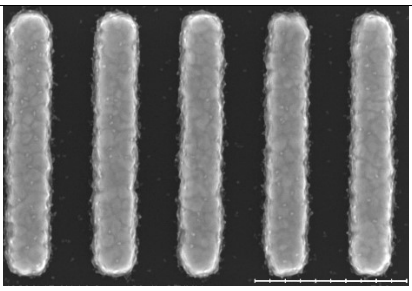	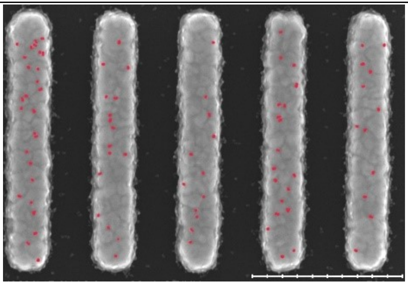
3	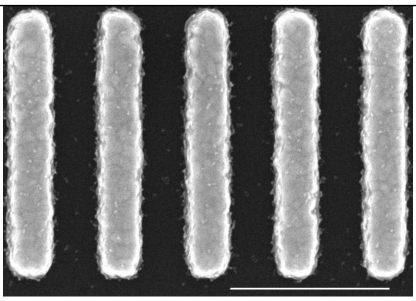	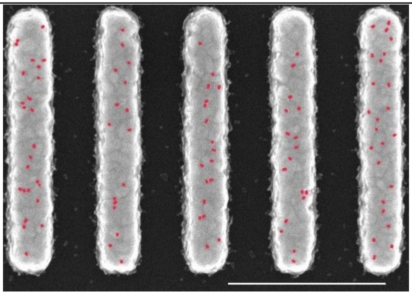
4	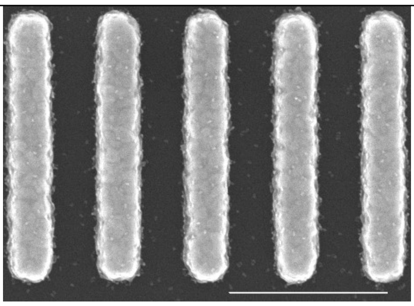	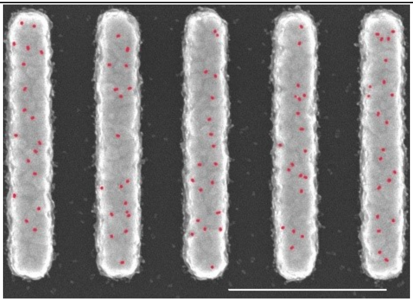

5	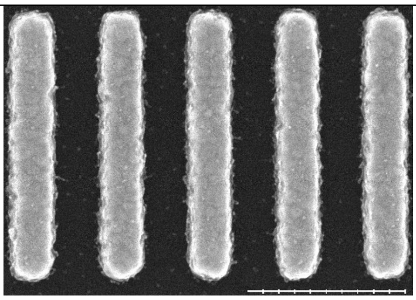	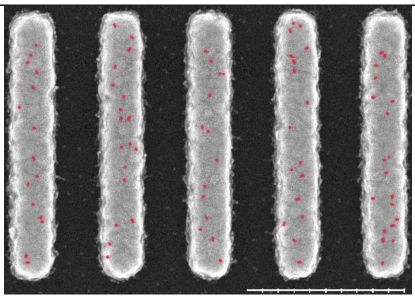
6	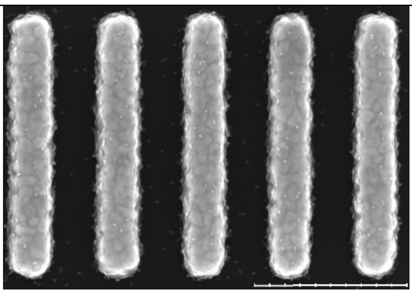	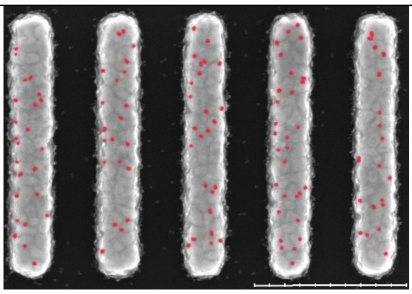

Long rods illuminated at 1000 nm / 90°		
#	Raw	Colorized
1	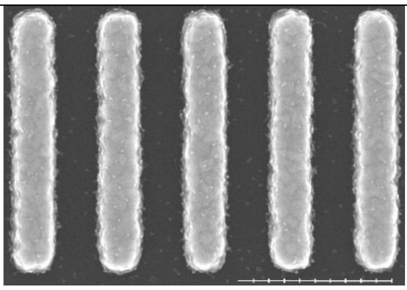	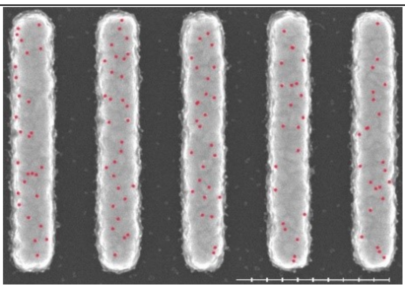
2	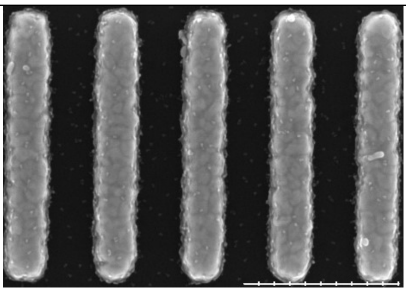	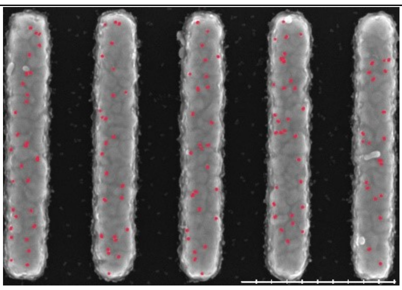
3	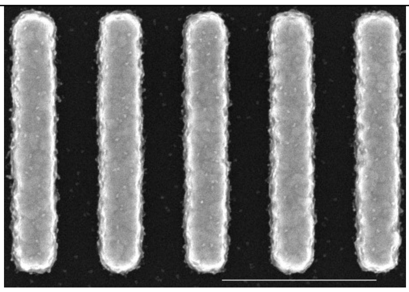	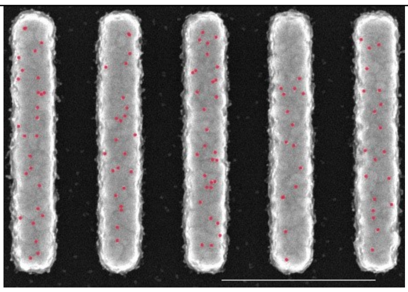

4	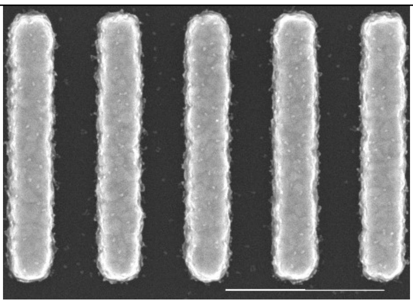	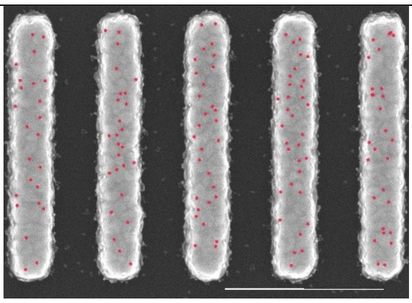
5	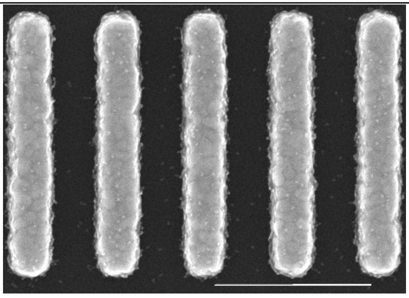	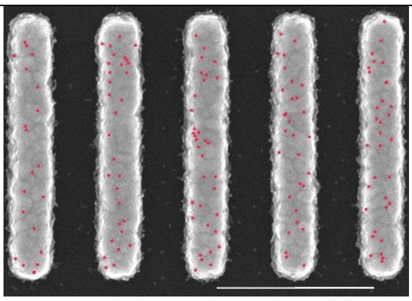
6	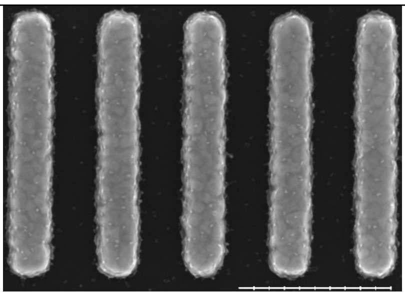	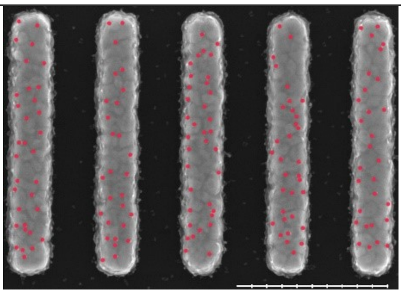

The number of QDs on each rod of the above section have been gathered in the tables below, the red numbers were excluded from the results as they were taken outside the middle part of the array (exposed to a lower intensity of the laser):

700 nm Unilluminated Summary										
**λ**	**SEM #**	**Rod 1**	**Rod 2**	**Rod 3**	**Rod 4**	**Rod 5**	**Average**	**SD**	**Average**	**SD**
**Unillu-minated**	**1**	23	28	28	22	23	**24.8**	**2.9**	**29.1**	**5**
	**2**	27	30	29	21	42	**29.8**	**7.7**		
	**3**	27	32	25	27	30	**28.2**	**2.8**		
	**4**	37	39	32	37	33	**35.6**	**3**		

	**5**	29	28	25	25	24	**26.2**	**2.2**		
	**6**	31	30	26	33	30	**30**	**2.5**		

700 nm 0° Summary										
**λ**	**SEM #**	**Rod 1**	**Rod 2**	**Rod 3**	**Rod 4**	**Rod 5**	**Average**	**SD**	**Average**	**SD**
**660 nm**	**1**	3	2	4	6	1	**3,2**	**1,9**	**6.3**	**3.3**
	**2**	9	11	11	5	5	**8,2**	**3,0**		
	**3**	11	5	7	5	9	**7,4**	**2,6**		
	**4**	7	2	3	7	4	**4,6**	**2,3**		
	**5**	4	8	7	2	6	**5,4**	**2,4**		
	**6**	12	9	14	5	5	**9**	**4,1**		
**690 nm**	**1**	2	3	5	8	8	**5,2**	**2,8**	**8.3**	**3.5**
	**2**	6	7	4	10	6	**6,6**	**2,2**		
	**3**	10	7	4	9	7	**7,4**	**2,3**		
	**4**	7	7	5	6	6	**6,2**	**0,8**		
	**5**	8	10	13	12	11	**10,8**	**1,9**		
	**6**	14	15	12	12	15	**13,6**	**1,5**		
**725 nm**	**1**	17	18	12	29	18	**18,8**	**6,2**	**21**	**5**
	**2**	21	19	21	25	17	**20,6**	**3,0**		
	**3**	14	24	20	14	20	**18,4**	**4,3**		
	**4**	18	12	19	20	22	**18,2**	**3,8**		
	**5**	26	26	24	19	23	**23,6**	**2,9**		
	**6**	24	27	21	25	34	**26,2**	**4,9**		

**840 nm**	**1**	17	20	21	19	18	**19**	**1,6**	**19.7**	**4.3**
	**2**	24	18	20	11	17	**18**	**4,7**		
	**3**	18	16	21	15	21	**18,2**	**2,8**		
	**4**	21	16	16	16	17	**17,2**	**2,2**		
	**5**	22	19	20	17	19	**19,4**	**1,8**		
	**6**	26	22	26	34	24	**26,4**	**4,6**		
**900 nm**	**1**	23	22	22	22	17	**21,2**	**2,4**	**22.9**	**5.4**
	**2**	21	24	17	19	28	**21,8**	**4,3**		
	**3**	30	17	23	21	20	**22,2**	**4,9**		
	**4**	18	18	22	19	20	**19,4**	**1,7**		
	**5**	22	20	17	21	22	**20,4**	**2,1**		
	**6**	30	31	35	36	31	**32,6**	**2,7**		

700 nm 90° Summary										
**λ**	**SEM #**	**Rod 1**	**Rod 2**	**Rod 3**	**Rod 4**	**Rod 5**	**Average**	**SD**	**Average**	**SD**
**660 nm**	**1**	5	6	7	5	7	**6**	**1,0**	**10.2**	**4.7**
	**2**	9	6	8	5	9	**7,4**	**1,8**		
	**3**	13	6	9	10	16	**10,8**	**3,8**		
	**4**	4	7	5	7	7	**6**	**1,4**		
	**5**	14	11	13	19	14	**14,2**	**2,9**		
	**6**	19	18	15	15	17	**16,8**	**1,8**		
**700 nm**	**1**	7	7	2	3	4	**4,6**	**2,3**	**9.6 (7.2)**	**4.5 (2.5)**

	**2**	8	4	7	5	9	**6,6**	**2,1**		
	**3**	9	8	7	5	6	**7**	**1,6**		
	**4**	9	9	11	14	10	**10,6**	**2,1**		
	**5**	14	11	14	16	8	**12,6**	**3,1**		
	**6**	17	15	16	17	17	**16,4**	**0,9**		
**840 nm**	**1**	11	18	18	11	12	**14**	**3,7**	**23.9**	**7.5**
	**2**	25	19	25	17	26	**22,4**	**4,1**		
	**3**	28	27	35	34	29	**30,6**	**3,6**		
	**4**	22	28	21	25	32	**25,6**	**4,5**		
	**5**	19	22	16	32	22	**22,2**	**6,0**		
	**6**	40	28	32	29	14	**28,6**	**9,4**		
**1000 nm**	**1**	14	19	21	19	17	**18**	**2,6**	**25.3**	**4.7**
	**2**	26	27	24	20	24	**24,2**	**2,7**		
	**3**	31	24	24	25	30	**26,8**	**3,4**		
	**4**	37	31	26	27	27	**29,6**	**4,6**		
	**5**	32	28	26	25	23	**26,8**	**3,4**		
	**6**	23	28	27	26	27	**26,2**	**1,9**		

800 nm *Unilluminated Summary*										
**λ**	**SEM #**	**Rod 1**	**Rod 2**	**Rod 3**	**Rod 4**	**Rod 5**	**Average**	**SD**	**Average**	**SD**
	**1**	36	30	31	34	31	**32.4**	**2.5**		
	**2**	28	28	24	32	23	**27**	**3.6**		
**Unillu-**	**3**	33	29	31	32	30	**31**	**1.6**	**32.6**	**6**
**minated**	**4**	25	30	26	27	29	**27.4**	**2.1**		
	**5**	32	34	39	38	40	**36.6**	**3.4**		
	**6**	39	35	50	43	39	**41.2**	**5.7**		

800 nm 0° Summary										
**λ**	**SEM #**	**Rod 1**	**Rod 2**	**Rod 3**	**Rod 4**	**Rod 5**	**Average**	**SD**	**Average**	**SD**
	**1**	1	1	0	4	3	**1,8**	**1,6**		
**660 nm**	**2**	3	3	0	1	2	**1,8**	**1,3**	**6.4**	**4.8**
	**3**	7	2	0	4	2	**3**	**2,6**		
	**4**	6	10	11	12	9	**9,6**	**2,3**		

	**5**	12	12	6	8	10	**9,6**	**2,6**		
	**6**	13	13	15	8	13	**12,4**	**2,6**		
**725 nm**	**1**	9	6	5	7	3	**6**	**2,2**	**9.6 (8)**	**4.8 (1.6)**
	**2**	3	8	7	7	12	**7,4**	**3,2**		
	**3**	11	10	7	10	9	**9,4**	**1,5**		
	**4**	6	5	11	4	11	**7,4**	**3,4**		
	**5**	8	9	11	13	9	**10**	**2,0**		
	**6**	21	11	22	21	12	**17,4**	**5,4**		
**790 nm**	**1**	13	8	8	16	17	**12,4**	**4,3**	**15.6**	**4**
	**2**	13	8	10	10	16	**11,4**	**3,1**		
	**3**	20	14	14	17	15	**16**	**2,5**		
	**4**	13	19	17	15	17	**16,2**	**2,3**		
	**5**	20	21	16	22	15	**18,8**	**3,1**		
	**6**	20	19	20	14	21	**18,8**	**2,8**		
**840 nm**	**1**	13	15	7	17	14	**13,2**	**3,8**	**16.3**	**5.3**
	**2**	14	17	8	8	15	**12,4**	**4,2**		
	**3**	16	11	12	9	13	**12,2**	**2,6**		
	**4**	16	19	12	20	24	**18,2**	**4,5**		
	**5**	24	25	20	13	17	**19,8**	**5,0**		
	**6**	23	20	20	22	26	**22,2**	**2,5**		
**900 nm**	**1**	27	18	24	22	24	**23**	**3,3**	**21.8**	**3.9**
	**2**	22	16	24	18	19	**19,8**	**3,2**		
	**3**	23	26	25	28	22	**24,8**	**2,4**		
	**4**	26	19	20	25	20	**22**	**3,2**		

	**5**	19	24	21	13	28	**21**	**5,6**		
	**6**	16	17	18	27	22	**20**	**4,5**		

800 nm 90° Summary										
**λ**	**SEM #**	**Rod 1**	**Rod 2**	**Rod 3**	**Rod 4**	**Rod 5**	**Average**	**SD**	**Average**	**SD**
**660 nm**	**1**	6	5	3	4	8	**5,2**	**1,9**	**11.9**	**5.6**
	**2**	6	8	8	11	5	**7,6**	**2,3**		
	**3**	16	10	6	8	14	**10,8**	**4,1**		
	**4**	20	10	13	15	12	**14**	**3,8**		
	**5**	16	12	12	14	13	**13,4**	**1,7**		
	**6**	19	22	22	18	21	**20,4**	**1,8**		
**700 nm**	**1**	11	13	20	18	12	**14,8**	**4,0**	**17.3**	**5.2**
	**2**	5	14	14	13	12	**11,6**	**3,8**		
	**3**	15	15	18	15	12	**15**	**2,1**		
	**4**	21	29	18	14	24	**21,2**	**5,7**		
	**5**	25	26	18	23	19	**22,2**	**3,6**		
	**6**	16	24	16	20	19	**19**	**3,3**		
**840 nm**	**1**	19	14	12	26	20	**18,2**	**5,5**	**19.2**	**4.5**
	**2**	27	16	12	19	12	**17,2**	**6,2**		

	**3**	25	16	18	17	24	**20**	**4,2**		
	**4**	18	18	19	19	21	**19**	**1,2**		
	**5**	15	18	15	17	19	**16,8**	**1,8**		
	**6**	24	21	25	30	19	**23,8**	**4,2**		
**1000 nm**	**1**	28	30	26	21	22	**25,4**	**3,8**	**27.2**	**5**
	**2**	29	26	27	30	20	**26,4**	**3,9**		
	**3**	26	22	30	14	20	**22,4**	**6,1**		
	**4**	24	27	30	33	26	**28**	**3,5**		
	**5**	20	31	34	29	32	**29,2**	**5,4**		
	**6**	33	28	35	33	29	**31,6**	**3,0**		

Nanoheating Experiment 10 mW

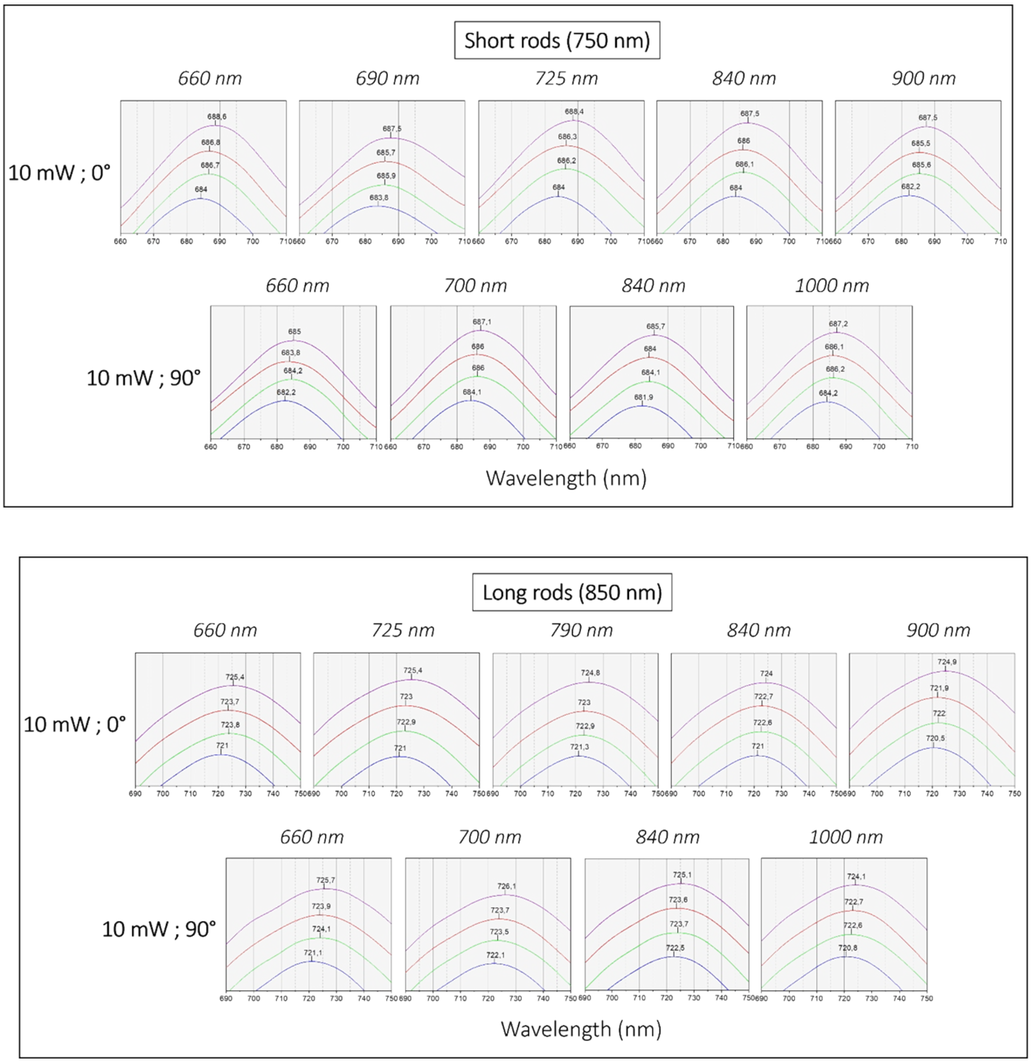

Figure S6: Reflectance spectra of the short rod arrays (top panel) and long rods (bottom panel) taken in water (blue), after *p-NIPAAM* deposition (green), following the 10 mW laser heating at the wavelength indicated (red) and finally after the QDs functionalization (purple).

Reflectance spectra:

Reflectance shift compared to the QDs reduction factor R:

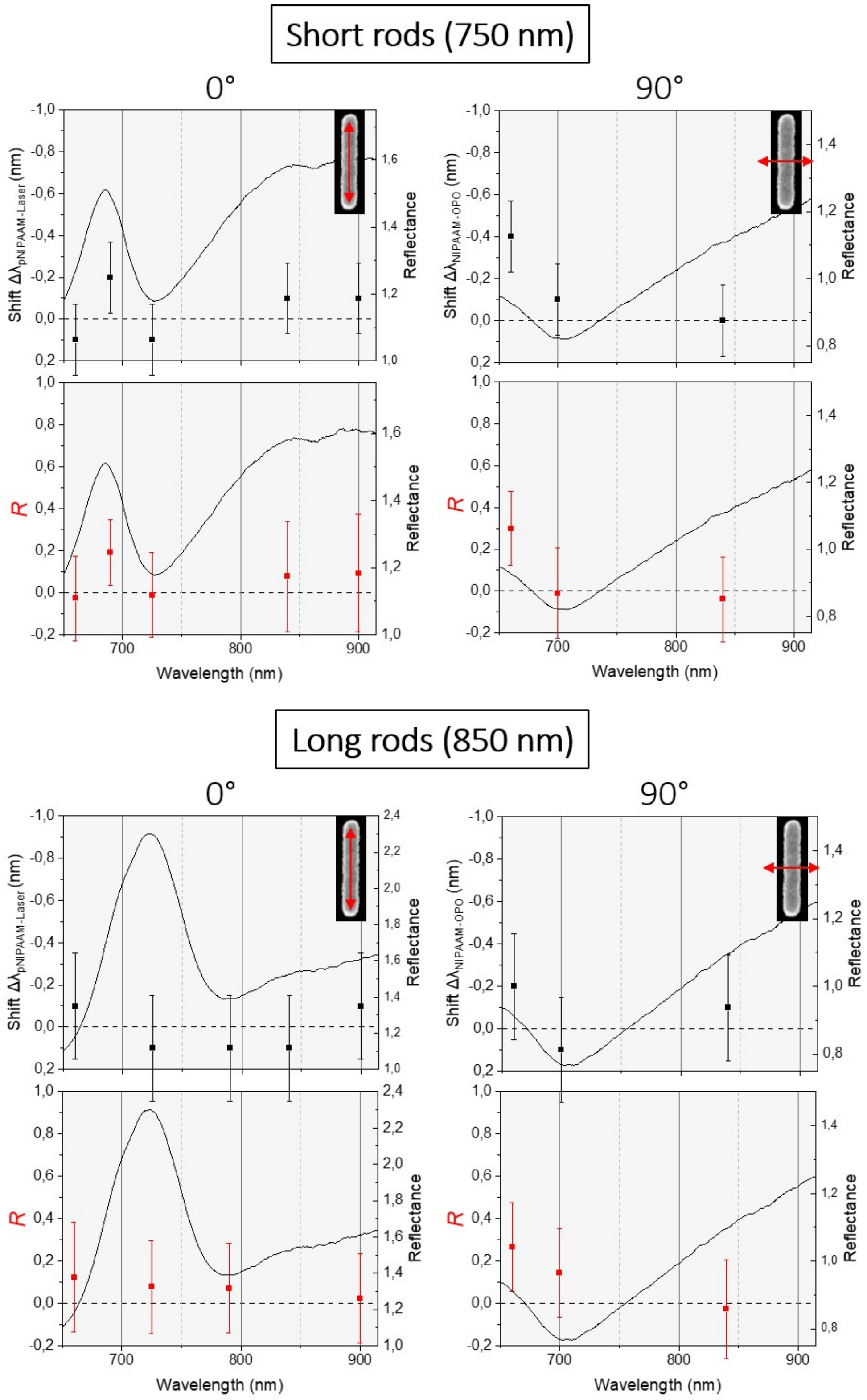

Figure S7: Short rods data (top panel) and long rods (bottom panel) of reflectance shift $\Delta\lambda_{pNIPAAM-Laser}$ as a function of the wavelength of the pulsed laser, for both 0° and 90° laser polarizations on the left and right column respectively (shown by the red arrow relative to a rod on the top right of each plot). The reflectance spectra corresponding to the laser polarization were overlayed on each panel. The error bar represents the standard deviation of 5 spectra. The R factor in red is relative to all the laser wavelengths used at both polarization angles. The error bar is the standard deviation from the QDs count of 30 different rods.

SEM images compared:

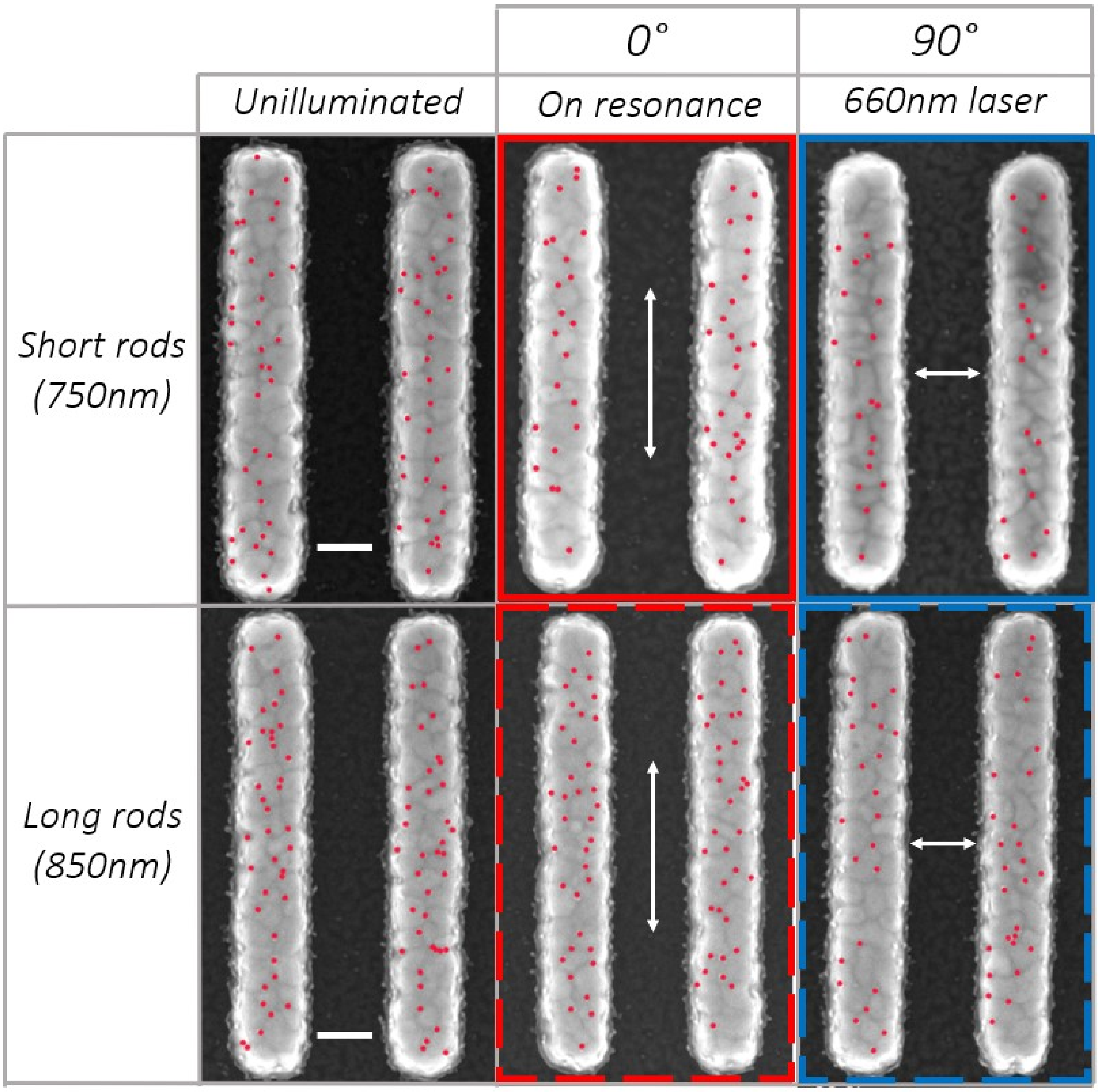

Figure S8: SEM images with colorized QDs in red of the 10 mW power experiment in the case where the rods were left unilluminated (left column), when heated by a laser at 0° on the plasmonic resonance (690 nm for the short rods, and 725 nm for the long rods). And on the right column, at 660 nm for a 90° angle.

Numerical Simulations

Finite-element simulations were performed using COMSOL Multiphysics v6.0 to model both the optical and thermal responses of the nanorod arrays. All geometries were reconstructed directly from AFM profiles, ensuring that nanorod curvature and tapering were faithfully represented in the models.

Optical model (Wave Optics)

The optical properties were simulated using the Wave Optics module. Unit-cell dimensions of 900 × 280 nm (short rods) and 1000 × 280 nm (long rods) were embedded in a 1600 nm-high computational domain. Periodic boundary conditions were applied laterally to reproduce the array, while perfectly matched layers (PMLs) were placed at the top and bottom of the domain to suppress spurious reflections.

Optical constants for gold were primarily taken from Johnson and Christy[2]. For comparison, simulations were also performed using Palik's optical constants, which gave qualitatively similar results. Meshing was optimised for accuracy: the PMLs were meshed with a swept scheme, while the nanorods and surrounding dielectric were discretised with tetrahedral elements, limited to 18 nm in gold and 30 nm in the dielectric.

Illumination was defined as a normally incident plane wave. For 0° polarisation, the electric field was aligned along the nanorod long axis; for 90° polarisation, it was aligned along the short axis.

The resulting reflectance spectra are shown in Figure S9, where simulated data for both rod lengths and polarisations are compared with experimental spectra. To align the simulated and measured resonance positions, the calculated spectra were blue-shifted by 20 nm.

To assess field localisation, the electric field intensity distribution was calculated for an 800 nm nanorod under resonant long-axis excitation. The results are presented in Figure S10, which shows both plan and cross-sectional views. These maps highlight the strong confinement of optical fields at the rod surface and their penetration into the surrounding aqueous medium, consistent with the regions where thermally induced polymer collapse and switching occur.

Thermal model (Heat Transfer)

The Heat Transfer module was used to simulate the transient heating of the nanorods under pulsed laser excitation. The same AFM-derived geometries were used as in the optical model. Heating was applied as a 5 ns square laser pulse, implemented as a time-dependent boundary condition, with illumination geometry matched to the optical simulations.

Material properties for gold, silicon, and water were taken from the COMSOL database. Temperature-dependent variations of optical and thermal constants were neglected. Heat transfer into the silicon substrate and the overlying water was included, with the water domain treated under steady-state boundary conditions (convective flow neglected).

This model provided estimates of the nanosecond-scale heating and cooling dynamics of the nanorods and the adjacent interfacial water layer, linking the optical absorption behaviour observed in Figure S9 and the localised near-field distributions shown in Figure S10 to the experimentally observed switching behaviour.

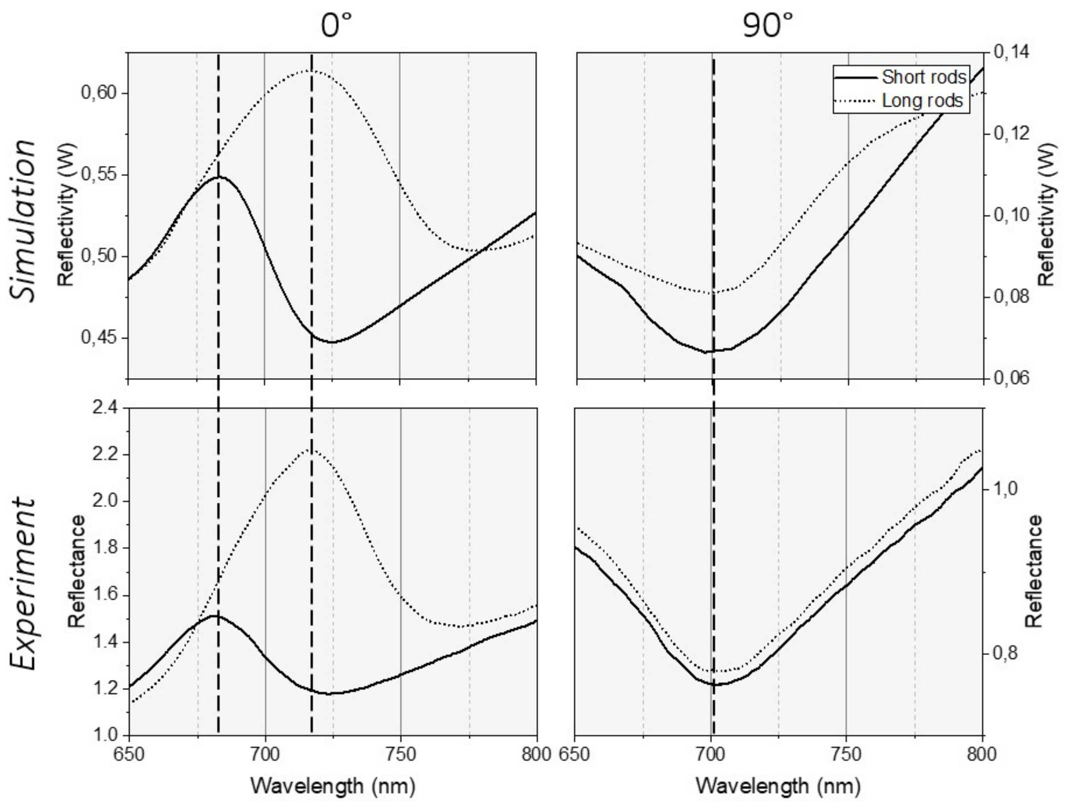

Figure S9: Simulated reflectivity spectra (top row) and experimental spectra (bottom row) for the 0° (left column) and 90° polarization (right column). With the short rod data in the dark solid line and the long rod data dashed. The position of the resonance peaks is highlighted by dashed vertical lines.

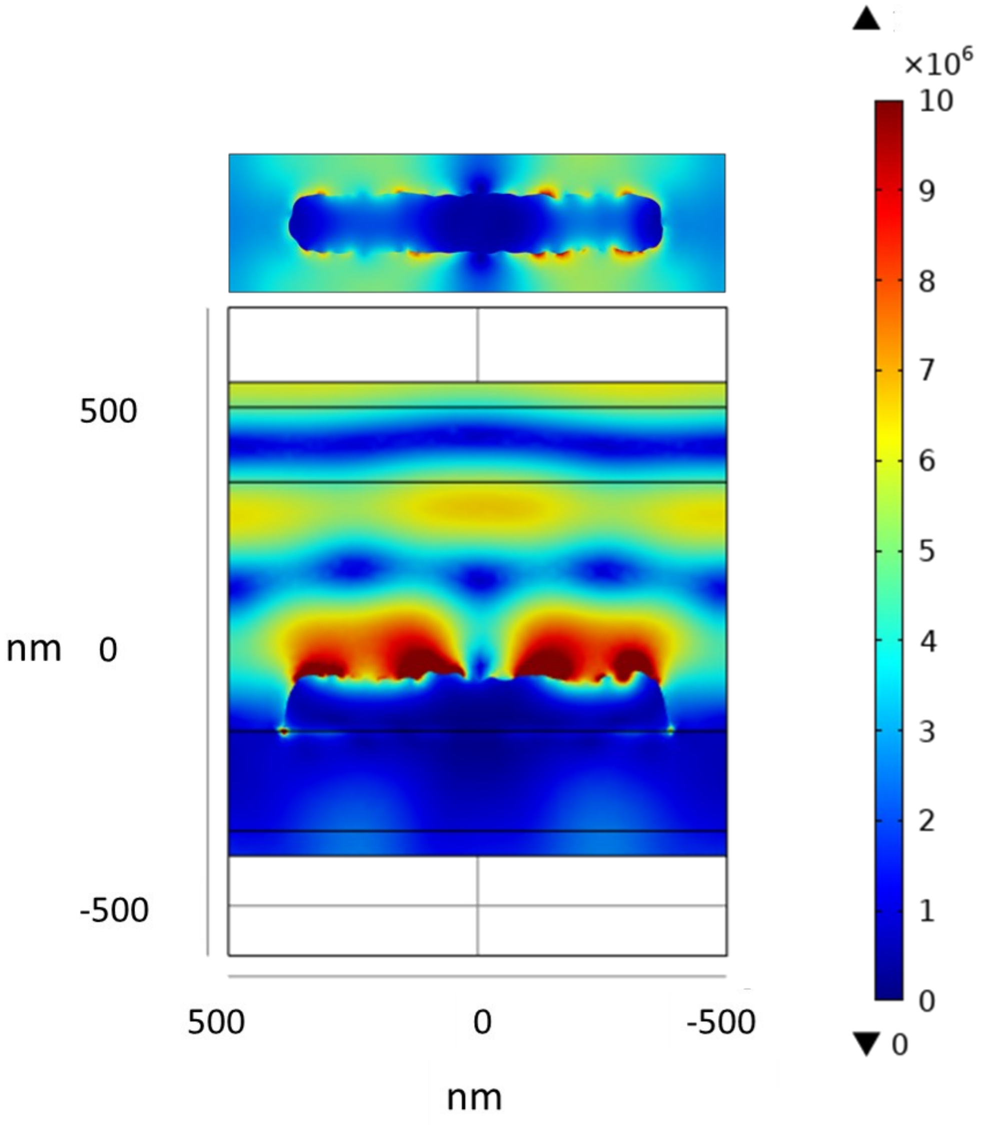

Figure S10. The spatial extent of near fields from a top and cross-sectional views for resonant excitation of 800 nm nano-rod along the long axis.

References

[1] Johnson, P. B.; Christy, R. W. Optical Constants of the Noble Metals. Phys. Rev. B 1972, **6** (12), 4370– 4379.

p-NIPAM characterization

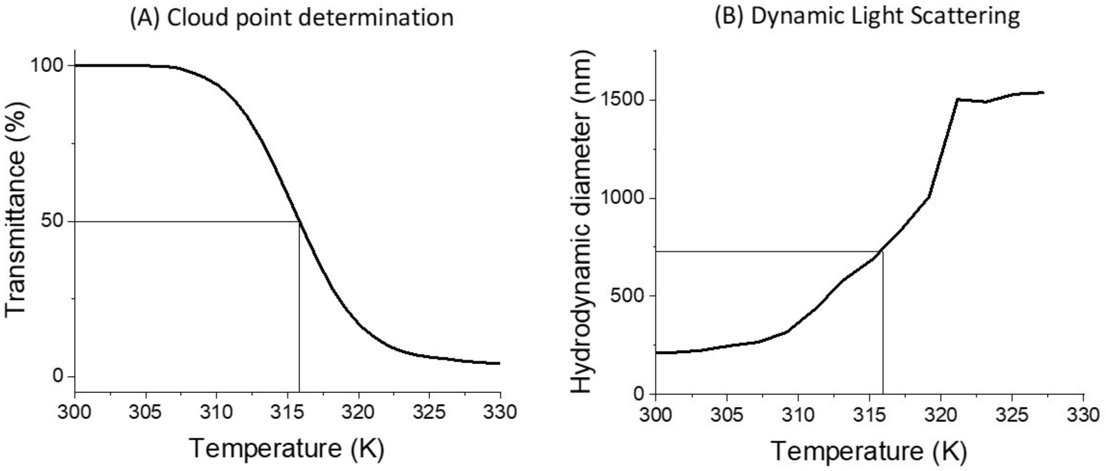

Figure S11. (A) Transmittance at 500 nm of *p-NIPAM* in water (0.2 mg/mL) as a function of the temperature and (B) dynamic light scattering analysis as a function of temperature. The curves were extrapolated from a set of experimental data points obtained at fixed temperatures. The LCST corresponds to the mid-point of the transmittance curve.

References

(1) Hajji, M.; Cariello, M.; Gilroy, C.; Kartau, M.; Syme, C. D.; Karimullah, A.; Gadegaard, N.; Malfait, A.; Woisel, P.; Cooke, G.; et al. Chiral Quantum Metamaterial for Hypersensitive Biomolecule Detection. *Acs Nano* **2021**, *15* (12), 19905-19916. DOI: 10.1021/acsnano.1c07408.
(2) Johnson, P. B.; Christy, R. W. Optical Constants of the Noble Metals. *Physical Review B* **1972**, *6* (12), 4370-4379. DOI: 10.1103/PhysRevB.6.4370.
